# Interactive effect of increased high sensitive C-reactive protein and dyslipidemia on cardiovascular diseases: a 12-year prospective cohort study

**DOI:** 10.1186/s12944-023-01836-w

**Published:** 2023-07-04

**Authors:** Solim Essomandan Clémence Bafei, Xianghai Zhao, Changying Chen, Junxiang Sun, Qian Zhuang, Xiangfeng Lu, Yanchun Chen, Xincheng Gu, Fangyuan Liu, Jialing Mu, Lai Wei, Pengfei Wei, Yunjie Yin, Hankun Xie, Song Yang, Chong Shen

**Affiliations:** 1 Department of Epidemiology, Center for Global Health, School of Public Health, Nanjing Medical University, Nanjing, 211166 China; 2 Department of Cardiology, Affiliated Yixing People’s Hospital of Jiangsu University, People’s Hospital of Yixing City, Yixing, 214200 China; 3 Department of Epidemiology, Fuwai Hospital, National Center for Cardiovascular Diseases, Chinese Academy of Medical Sciences and Peking Union Medical College, Beijing, China; 4 Key Laboratory of Cardiovascular Epidemiology, Chinese Academy of Medical Sciences, Beijing, China; 5 Research Units of Cohort Study on Cardiovascular Diseases and Cancers, Chinese Academy of Medical Sciences, Beijing, China

**Keywords:** hs-CRP, Dyslipidemia, Cardiovascular diseases risk, Interactive effect

## Abstract

**Background:**

Dyslipidemia and inflammation are significant factors for the onset of cardiovascular diseases (CVD); however, studies regarding their interactions on the risk of CVD are scarce. This study aimed to assess the interaction of dyslipidemia and high-sensitivity C-reactive protein (hs-CRP) on CVD.

**Methods:**

This prospective cohort enrolled 4,128 adults at baseline in 2009 and followed them up until May 2022 for collecting CVD events. Cox-proportional hazard regression analysis estimated the hazard ratios (HRs) and 95% confidence intervals (CIs) of the associations of increased hs-CRP (≥ 1 mg/L) and dyslipidemia with CVD. The additive interactions were explored using the relative excess risk of interaction (RERI) and the multiplicative interactions were assessed with *HRs* (95% *CI*) while the multiplicative interactions were assessed by the HRs (95% CI) of interaction terms.

**Results:**

The HRs of the association between increased hs-CRP and CVD were 1.42 (95% *CI*: 1.14–1.79) and 1.17 (95% *CI*: 0.89–1.53) among subjects with normal lipid levels and subjects with dyslipidemia, respectively. Stratified analyses by hs-CRP levels showed that among participants with normal hs-CRP (< 1 mg/L), TC ≥ 240 mg/dL, LDL-C ≥ 160 mg/dL, non-HDL-C ≥ 190 mg/dL, ApoB < 0.7 g/L, and LDL/HDL-C ≥ 2.02 were associated with CVD [*HRs* (95%*CIs*): 1.75 (1.21–2.54), 2.16 (1.37–3.41), 1.95 (1.29–2.97), 1.37 (1.01–1.67), and 1.30 (1.00-1.69), all *P* < 0.05, respectively]. While in the population with increased hs-CRP, only ApoAI > 2.10 g/L had a significant association with CVD [*HR* (95% CI): 1.69 (1.14–2.51)]. Interaction analyses showed that increased hs-CRP had multiplicative and additive interactions with LDL-C ≥ 160 mg/dL and non-HDL-C ≥ 190 mg/dL on the risk of CVD [*HRs* (95%*CIs*): 0.309 (0.153–0.621), and 0.505 (0.295–0.866); RERIs (95%*CIs*): -1.704 (-3.430-0.021 and − 0.694 (-1.476-0.089), respectively, all *P* < 0.05].

**Conclusion:**

Overall our findings indicate negative interactions between abnormal blood lipid levels and hs-CRP on the risk of CVD. Further large-scale cohort studies with trajectories measurement of lipids and hs-CRP might verify our results as well explore the biological mechanism behind that interaction.

**Supplementary Information:**

The online version contains supplementary material available at 10.1186/s12944-023-01836-w.

## Introduction

Cardiovascular diseases (CVD) continue to be a leading factor in early death and rising disability and healthcare expenses [1]. In China, over 40% of deaths are related to CVD [1] and the number of people with CVD rose from 50 million in 1990 to 120 million in 2019 and the number of death related to CVD almost doubled [2]. The higher burden of CVD makes it imperative to enhance their diagnostic and therapeutic capacities as well as preventive methods [3].

Common risk factors of CVD include dyslipidemia, hypertension, physical inactivity, diabetes, smoking, unhealthy diet, and alcohol abuse, which tend to co-exist and interact with each other to increase the risk of CVD [4, 5]. Dyslipidemia and low-grade inflammation are known as key drivers of atherosclerosis which is a pathological condition involved in the onset and progression of most CVD including stroke and CHD [6]. Abnormal levels of blood lipids are known as dyslipidemia and the role of abnormal levels of traditional lipids such as high-density lipoprotein cholesterol (HDL-C), total cholesterol (TC), low-density lipoprotein cholesterol (LDL-C), and triglycerides (TG) in the onset and progression of CVD has been extensively documented [7–10]. In addition, recent studies also showed that abnormal levels of non-conventional lipids such as TC/HDL-C, LDL-C/HDL-C, TG/HDL-C, non-HDL-C, apolipoprotein AI (ApoAI), apolipoprotein B (ApoB), and lipoprotein(a) [Lp(a)] are also performing indicators of the risk of CVD and cardiovascular events [11, 12].

Low-grade inflammation is characterized by a slight chronic elevation of inflammatory markers in the blood, but not to the same degree as acute inflammation [13]. High-sensitivity C-reactive protein (hs-CRP), known as a classic indicator for low-grade inflammation [14], has undergone extensive research among diverse groups of inflammatory biomarkers and has drawn the greatest attention because of its potential as a reliable and affordable predictor for CVD [3], regardless of lipids levels [15]. Hs-CRP is an acute-phase protein stimulated by proinflammatory cytokines [16] produced by hepatic aortic endothelial cells and coronary artery smooth muscle cells under oxidative stress or inflammatory stimulation [17, 18]. Previous studies demonstrated that higher levels of hs-CRP contributed to CVD incidence [19–21], recurrence [22], and mortality [23]. As per reports from the American Heart Association, hs-CRP less than 1, 1–3, and over 3 mg/L are considered low, medium, and high risk of CVD, respectively based on the Western population data [24]. However, Asians have low levels of hs-CRP with a median below 1 mg/L [25], and our previous cohort study indicated that the hs-CRP cut-off point of 1 mg/L was appropriate for ischemic stroke prediction in a Chinese population [26].

Accumulating evidence has shown that dyslipidemia and increased hs-CRP are factors associated with CVD and abnormal lipid levels are often related to abnormal levels of inflammatory biomarkers including hs-CRP [27, 28]. Nonetheless, studies regarding the potential interaction between hs-CRP and dyslipidemia are scant. Therefore, this study aimed to explore the interaction between dyslipidemia and increased hs-CRP levels on the risk of CVD in a 12-years prospective cohort of the Chinese population.

## Methods

### Study design and population

This prospective cohort study involved 4,128 adults aged 19 to 96 from Yixing City in China. A stratified cluster sampling method was used to select 5400 subjects over 18 years old from 6 villages of two townships of Yixing City, Jiangsu Province, China. Participants were included in our study based on the following criteria: (1) be aged 18 years or above at the time of the survey and (2) consent to take part in the study. A total of 4175 people participated in the baseline survey in May 2009, giving a response rate of 77.3%; among them, 4128 (98.9%) with complete data were included in this cohort study. Using the local disease and death register system of the Centers for Disease Control and Prevention (CDC), the outcomes events were recorded every year until May 2022. We have excluded 30 subjects with baseline stroke, 50 subjects with baseline CHD, and 78 subjects with baseline CVD from the corresponding analyses. Each participant signed an informed consent form to take part in the study. Nanjing Medical University’s ethics committee approved our research protocol (#200803307).

### Data collection and definitions of covariates

All subjects underwent physical examinations and laboratory testing following their interviews. The demographic characteristics of the study subjects were acquired using a standardized questionnaire. Subjects who smoked at least 20 cigarettes weekly for a trimester or more in a year were classified as smokers. Alcohol drinkers were those who currently or previously consumed alcohol at least two times weekly for a semester or more in a year. Each study subject’s weight, height, and thrice blood pressure were measured using standardized instruments. Body mass index (BMI) was calculated by dividing the weight (kg) by the height squared (m^2^). Subjects who had average systolic blood pressure (SBP) of 140 mmHg and above or diastolic blood pressure (DBP) of 90 mmHg and above, a self-reported history of hypertension, or who were actively taking an antihypertensive drug were classified as hypertensive. 

We used the Olympus AU2700 automatic biochemistry analyzer to evaluate TC, HDL-C, TG, LDL-C, ApoB, ApoAI, Lp(a), and glucose of the plasma after overnight fast over eight hours. A high-sensitivity immunoturbidimetric test measured hs-CRP levels. The range for normal hs-CRP levels was set as less than 1 mg/L while hs-CRP ≥ 1 mg/L was recognized as increased levels [26]. Diabetes cases were classified as subjects whose fasting plasma glucose (FPG) was 126 mg/dL or above, who self-reported having had the disease in the past, or who were taking hypoglycemic medication at the time.

The non-HDL-C was obtained by subtracting HDL-C from TC and its cut-off point was 190 mg/dL [29]. TC, TG, LDL-C, and HDL-C’s cut-off points were 240 mg/dL, 200 mg/dL, 160 mg/dL, and 40 mg/dL respectively [29]. Dyslipidemia cases had TC ≥ 240 mg/dL, TG ≥ 200 mg/dL, LDL-C ≥ 160 mg/dL, HDL-C < 40 mg/dL, self-reported having dyslipidemia or used actively lipid-lowering medicines (n = 19) as recommended by the 2016 Chinese Adults dyslipidemia Prevention guidelines [29]. The medians of TG/HDL-C, TC/HDL-C, and LDLC/HDL-C were used as cut-off points (2.27, 3.64, and 2.02, respectively) because they have no clear clinical diagnostic standards. The cut-off points of ApoAI, ApoB, and Lp(a) were set to 1.6–2.1 g/L, 0.70–0.90 g/L, and 90 mg/L respectively based on our previous study results which indicate that those cut-off points were appropriate for CVD prediction [30].

### Measurement of outcomes

CVD events were identified based on records of the Center for Disease Control and Prevention, followed by further examination by cardiologists and neurologists. CVD events in this study comprised stroke and CHD. Stroke and CHD were identified according to the International Classification of Diseases, Tenth Revision, and Clinical Modification (ICD-10-CM) code.

### Statistical analysis

Before the main analyses, the distributions of continuous parameters were examined. Then the medians and interquartile ranges (*IQR*) were calculated for continuous parameters with non-normal distribution, and the Kruskal-Wallis *H* test explored their differences among hs-CRP and dyslipidemia groups. Frequencies were calculated for categorical variables, and their differences among hs-CRP and dyslipidemia groups were assessed using Chi-square (*χ*^*2*^) test. Cox proportional hazard regression models estimated the hazard ratios (*HRs*) and 95% confidence interval (95% *CI*) of the association of lipids and hs-CRP with CVD after adjustment of confounding factors. The models were tested and plotted based on scaled Schoenfeld residuals to explore that proportional hazards were not violated. Then, the Cochrane Q test was performed to explore statistical heterogeneity among subgroups. Multiple restricted cubic splines (RCS) analyses were performed to investigate the linearity between lipids and CVD in the subgroups of hs-CRP. Additive interactions were explored using the relative excess risk of interaction (RERI) and attributable proportion (AP). The RERI or AP > 0 statistically significantly indicates a positive interaction and below 0 indicates a negative interaction. The multiplicative interactions were assessed by the *HRs* (95% *CI*) of interaction terms, with the estimated value significantly below 1 indicating a negative interaction and greater than 1 indicating a positive interaction [31].

We also performed sensitivity analyses to evaluate the association of hs-CRP and CVD after excluding individuals with lipid-lowering treatment (n = 19), and the interaction of abnormal lipid levels and increased hs-CRP using another two cut-off points of 3 mg/L and 6 mg/L. Statistical significance was established as a two-tailed with *P* below 0.05. The analyses were conducted in SAS version 9.4 and R-studio version 4.2.1.

## Results

### The features of the study population based on lipid and hs-CRP levels

This study included 4,128 subjects with a median age of 58.95 (IQR: 52.24, 67.00) and 2444 (59.21%) women (Table 1). The subjects with both dyslipidemia and increased hs-CRP (18.73%) had relatively higher medians of age, blood pressure, BMI, TC, TG, LDL-C, ApoAI, ApoB, and Lp(a) and a lower median of HDL-C and higher proportions of hypertension and diabetes than subjects with normal lipids and normal hs-CRP (38.42%).

**Table 1:** Clinical characteristics of study subjects by hs-CRP and lipids levels

Variables	All subjects *N* = 4128	Normal lipids and normal hs-CRP *n* = 1586 (38.42%)	Dyslipidemia and normal hs-CRP *n* = 716 (17.34%)	Normal lipids and increased hs-CRP *n* = 1053 (25.51%)	Dyslipidemia and increased hs-CRP *n* = 773 (18.73%)	H/χ^2^	*P*
Age (years)	58.95 (52.24, 67.00)	57.26 (51.00, 63.99)	56.81 (50.08, 64.88)	61.85 (55.24, 71.04)	61.01 (54.10, 70.37)	181.22	< 0.001*
Gender *n* (%)							
Women	2444 (59.21)	894 (55.37)	404 (56.42)	652 (61.92)	494 (63.91)	17.86	< 0.001*
Men	1684 (40.79)	692 (43.63)	312 (43.58)	401 (38.08)	279 (36.09)		
Smoking *n* (%)							
Yes	1005 (24.35)	428 (26.99)	177 (24.72)	238 (22.60)	162 (21.96)	12.62	0.006*
No	3123 (75.65)	1158 (73.01)	539 (75.28)	815 (77.40)	611 (79.04)		
Drinking *n* (%)							
Yes	891 (21.58)	371 (23.39)	161 (22.49)	213 (20.22)	146 (18.89)		
No	3237 (78.42)	1215 (76.61)	555 (77.51)	840 (79.77)	627 (81.11)	7.87	0.049*
Hypertension *n* (%)							
Yes	2015 (48.81)	642 (40.48)	366 (51.12)	555 (52.71)	452 (58.47)	80.87	< 0.001*
No	2113 (51.19)	944 (59.52)	350 (48.88)	498 (47.29)	321 (41.53)		
DM *n* (%)							
Yes	468 (11.34)	109 (6.87)	107 (14.94)	113 (10.73)	139 (17.98)	75.05	< 0.001*
No	3660 (88.66)	1477 (93.13)	609 (85.06)	940 (89.27)	634 (82.02)		
Overweight/Obese *n* (%)							
Yes	2072 (50.19)	608 (38.34)	375 (52.37)	568 (53.94)	521 (67.40)	188.03	< 0.001*
No	2056 (49.81)	978 (61.66)	241 (47.63	485 (46.06)	252 (32.60)		
SBP (mmHg)	134 (123, 14)	131 (120, 140)	134 (123, 143)	135 (125, 141.5)	137 (127, 145)	56.75	< 0.001*
DBP (mmHg)	82 (78, 89)	81 (78, 88)	83 (79, 90)	82 (78, 89)	83 (79, 89)	27.79	< 0.001*
BMI (kg/m^2^)	24.01 (21.93, 26.42)	23.00 (21.14, 25.30)	24.23 (22.38, 26.40)	24.44 (22.04, 26.71)	25.65 (23.19, 27.91)	280.60	< 0.001*
HDL-C (mg/dL)	51.35 (43.63, 59.85)	54.05 (47.10, 61.40)	43.24 (37.07, 56.37)	53.67 (47.30, 61.39)	44.10 (37.45, 57.53)	483.24	< 0.001*
LDL-C(mg/dL)	102.32 (84.94, 120.08)	99.61 (85.33, 115, 06)	103.47 (79.54, 128.96)	104.25 (88.80, 119.30)	105.02 (83.01, 131.85)	28.31	< 0.001*
Non-HDL-C (mg/dL)	133.59 (111.68, 157.14)	124.32 (104, 63, 144.40)	147.49 (119, 79, 179, 44)	130.12 (110, 42, 148.65)	154.05 (124.52, 183.78)	445.63	< 0.001*
TC (mg/dL)	185.33 (162.93, 210.42)	179.92 (161.62, 201.16)	193.44 (161.97, 236.97)	183.78 (167.06, 204.63)	200.00 (167.57, 240.93)	168.56	< 0.001*
TG (mg/dL)	116.81 (79.65, 176.99)	91.15 (67.26, 124.78)	202.66 (117.92, 278.32)	100.89 (73.45, 137.61)	217.70 (144.25, 285.84)	1335.55	< 0.001*
ApoAI (g/L)	1.57 (1.38, 1.80)	1.62 (1.44, 1.84)	1.49 (1.30, 1.73)	1.61 (1.43, 1.82)	1.49 (1.31, 1.72)	140.34	< 0.001*
ApoB (g/L)	0.90 (0.75, 1.08)	0.86 (0.73, 1.02)	0.97 (0.77, 1.16)	0.88 (0.75, 1.04)	1.00 (0.80, 1.18)	155.79	< 0.001*
Lp(a) (mg/L)	87.80 (43.75, 174.68)	86.78 (44.40, 159.03)	79.88 (36.53, 183.25)	94.00 (48.75, 185.65)	89.10 (42.95, 185.00)	13.14	0.004*
TC/HDL-C	3.64 (3.07, 4.22)	3.29 (2.84, 3.76)	4.22 (3.67, 4.89)	3.42 (2.93, 3.92)	4.33 (3.70, 5.00)	1082.97	< 0.001*
TG/HDL-C	2.27 (1.44, 3.74)	1.68 (1.18, 2.39)	4.17 (2.65, 6.39)	1.89 (1.29, 2.65)	4.62 (3.22, 6.59)	1559.64	< 0.001*
LDL-C/HDL-C	2.02 (1.64, 2.39)	1.84 (1.52, 2.17)	2.28 (1.87, 2.73)	1.95 (1.56, 2.29)	2.29 (1.92, 2.79)	506.78	< 0.001*

Notes: *: significance at 0.05. Normal hs-CRP: hs-CRP < 1 mg/L, increased hs-CRP: hs-CRP ≥ 1 mg/L. DBP: diastolic blood pressure, SBP: systolic blood pressure, TG: triglycerides; TC: total cholesterol, HDL-C: high-density lipoprotein cholesterol, LDL-C: low-density lipoprotein cholesterol, BMI: body mass index, ApoAI: apolipoprotein AI, ApoB: apolipoprotein B, Lp(a): lipoprotein(a). Values are presented as *M*: *median* (*IQR*: *interquartile range*) or *n (%).* For each quantitative variable, the *P*-value is obtained by the Kruskal Wallis *H* test; for each categorical variable, the *P*-value is obtained through Pearson’s χ^2^-test

### Association analysis of hs-CRP and dyslipidemia with CVD

There were 567 CVD cases reported throughout the follow-up duration with a median of 12.59 years, including 407 cases of stroke and 241 cases of CHD. The overall CVD incidence density was 117.30 per 10,000 person-years and subjects with both dyslipidemia and increased hs-CRP had the highest incidence density of CVD (169.59 per 10,000). The *HRs* of increased hs-CRP with stroke and CVD were 1.25 (95% *CI*: 1.03–1.54) and 1.30 (95% *CI*: 1.09–1.54) in the overall population. In the stratified model by dyslipidemia status, increased hs-CRP was significantly related to a higher risk of stroke [*HR* (*95%CI*): 1.34 (1.03–1.74), *P* = 0.030], and CVD [*HR* (*95%CI*): 1.42 (1.14–1.79), *P* = 0.002] among subjects with normal lipid levels, not among subjects with dyslipidemia (*P* > 0.05) (Table 2). In the stratified analysis by age group, increased hs-CRP was significantly associated with an increased hazard of stroke and CVD among subjects aged < 60 years [*HR* (95% *CI*): 2.18 (1.37–3.45) and 1.67 (1.16–2.39); all *P* < 0.05], not among subjects aged ≥ 60 years (*P* > 0.05) (Supplementary Table 1). Subjects aged < 60 years with normal lipid levels were 3.06 and 2.50 times at risk of stroke and CVD when their hs-CRP increased than subjects with dyslipidemia of the same age group. However, hs-CRP contributed to similar risks of CVD among subjects aged ≥ 60 years with dyslipidemia and not [*HR* (*95%CI*): 1.25 (0.91–1.71) and 1.28 (1.00-1.65), *P* ≥ 0.05, respectively].

There were significant associations of TC ≥ 240 mg/dL, non-HDL-C ≥ 190 mg/dL, and ApoAI > 2.10 g/L with CVD [*HR* (95%*CI*): 1.34 (1.06–1.72), 1.34 (1.02–1.75), and 1.44 (1.05–1.97), all *P* < 0.05, respectively] in the overall study population (Supplementary Table 2).

**Table 2:** Cox regression analysis for the association of increased hs-CRP with stroke, CHD, and CVD

Outcome	Exposure group	Incidence cases	Person-years	Incidence density (/10^4^ person-years)	*HR* (95% *CI*)	*P*
Stroke						
	Overall					
	hs-CRP < 1 mg/L	171	28400.96	60.21	Ref	
	hs-CRP ≥ 1 mg/L	236	21631.78	109.10	1.25 (1.03–1.54)	0.030*
	Dyslipidemia					
	Yes					
	hs-CRP < 1 mg/L	63	8788.92	71.68	Ref	
	hs-CRP ≥ 1 mg/L	101	9108.70	110.88	1.19 (0.86–1.66)	0.296
	No					
	hs-CRP < 1 mg/L	108	19612.03	55.07	Ref	
	hs-CRP ≥ 1 mg/L	135	12523.08	107.80	1.34 (1.03–1.74)	0.030*
	*P* for heterogeneity					0.583
CHD						
	Overall					
	hs-CRP < 1 mg/L	103	28574.96	36.05	Ref	
	hs-CRP ≥ 1 mg/L	138	22005.62	67.71	1.25 (0.96–1.64)	0.098
	Dyslipidemia					
	Yes	47	8772.85	53.57	Ref	
	hs-CRP < 1 mg/L	63	9353.41	67.36	1.06 (0.72–1.58)	0.759
	hs-CRP ≥ 1 mg/L					
	No					
	hs-CRP < 1 mg/L	56	19802.11	28.28	Ref	
	hs-CRP ≥ 1 mg/L	75	12652.21	59.28	1.41 (0.99–2.02)	0.058
	*P* for heterogeneity					0.307
CVD						
	Overall					
	hs-CRP < 1 mg/L	240	27732.35	86.54	Ref	
	hs-CRP ≥ 1 mg/L	327	20607.06	158.68	1.30 (1.09–1.54)	0.004*
	Dyslipidemia					
	Yes					
	hs-CRP < 1 mg/L	98	8437.70	116.15	Ref	
	hs-CRP ≥ 1 mg/L	147	8667.95	169.59	1.17 (0.89–1.53)	0.255
	No					
	hs-CRP < 1 mg/L	142	19294.65	73.60	Ref	
	hs-CRP ≥ 1 mg/L	180	11939.11	150.77	1.42 (1.14–1.79)	0.002*
	*P* for heterogeneity					0.283

Notes: *: Significance at 0.05. The model was adjusted for age, gender, smoking, drinking, hypertension, BMI, and diabetes. BMI: body mass index; CVD: cardiovascular disease, CHD: coronary heart disease, hs-CRP: high-sensitivity C-reactive protein. *HR*: hazard ratio, *CI*: confidence intervals

### Association between lipids and CVD stratified by hs-CRP levels

**Fig. 1:**
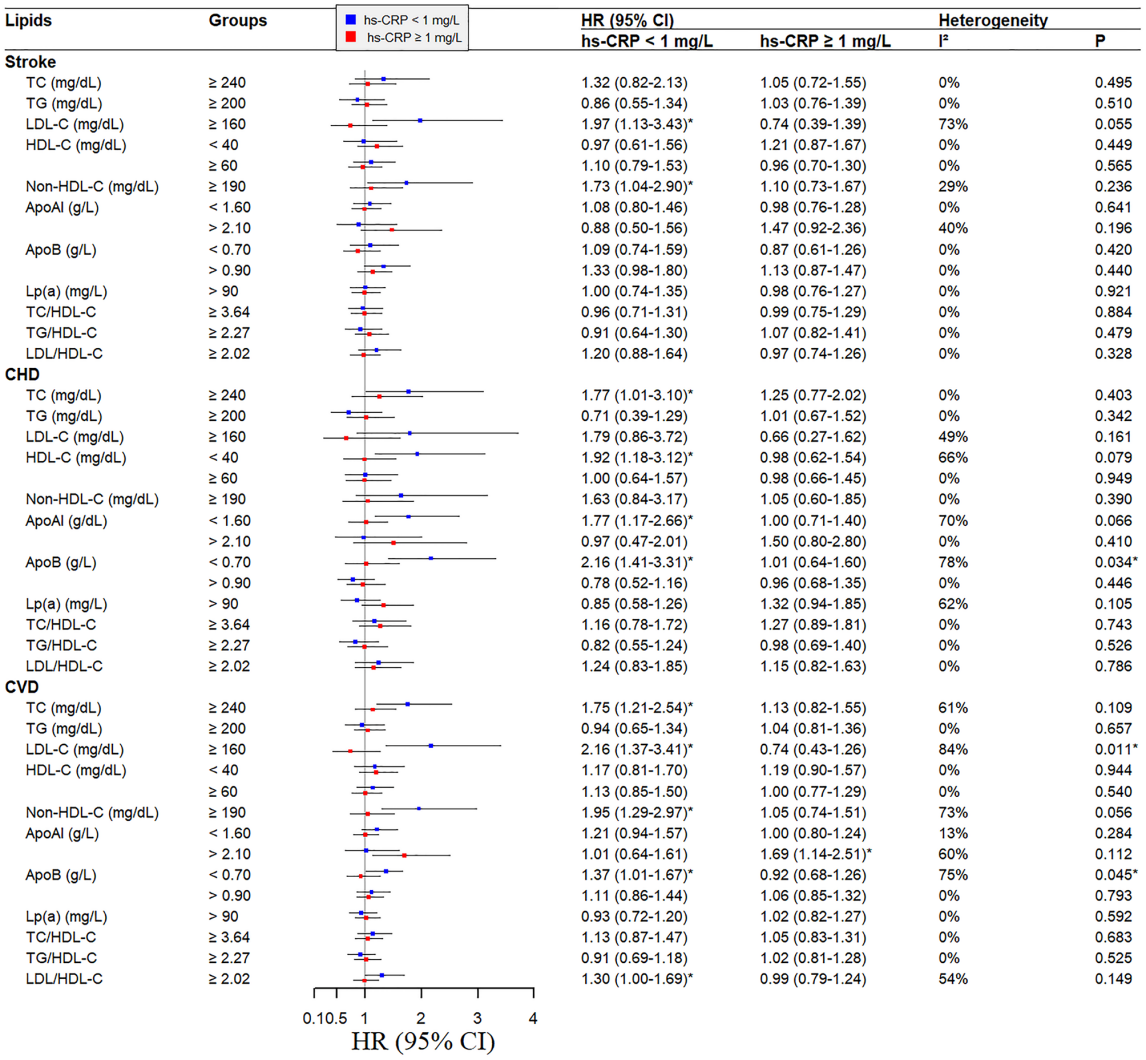
Multivariate Cox-regression of lipids and the risk of stroke, CHD, and CVD stratified by hs-CRP levels Notes: *: Significance at 0.05. The statistical significance for the heterogeneity test was set at *P* < 0.1. The model was adjusted for age, gender, smoking, drinking, hypertension, BMI, and diabetes. BMI: body mass index. Hs-CRP: high sensitivity C-reactive Protein, CHD: coronary heart disease, CVD: cardiovascular disease, TC: total cholesterol, TG: triglycerides, HDL-C: high-density lipoprotein cholesterol, LDL-C: low-density lipoprotein cholesterol, BMI: body mass index, ApoAI: apolipoprotein AI, ApoB: apolipoprotein B, Lp(a): lipoprotein(a), *HR*: hazard ratio, *CI*: confidence intervals

In the population with normal hs-CRP, there were significant associations of LDL-C ≥ 160 mg/dL and non-HDL-C ≥ 190 mg/dL with stroke [*HRs* (95%*CIs*): 1.97 (1.13–3.43) and 1.73 (1.04–2.90)], TC ≥ 240 mg/dL, HDL-C < 40 mg/dL, ApoAI < 1.60 g/L, and ApoB < 0.7 g/L were related to CHD [*HRs* (95%*CIs*): 1.77 (1.01–3.10), 1.92 (1.18–3.12), 1.77 (1.17–2.66), and 2.16 (1.41–3.31), respectively; all *P* < 0.05], and TC ≥ 240 mg/dL, LDL-C ≥ 160 mg/dL, non-HDL-C ≥ 190 mg/dL, ApoB < 0.7 g/L, and LDL/HDL-C ≥ 2.02 were related to CVD [*HRs* (95%*CIs*): 1.75 (1.21–2.54), 2.16 (1.37–3.41), 1.95 (1.29–2.97), 1.37 (1.01–1.67), and 1.30 (1.00-1.69), respectively; all *P* < 0.05]. While in the population with increased hs-CRP, ApoAI > 2.10 g/L had a significant association with CVD [*HR* (95% CI): 1.69 (1.14–2.51); *P* < 0.05] (Fig. 1).

We further explored the statistical heterogeneity of the associations of lipids with CVD in subgroups of normal and increased hs-CRP. We found that the association of LDL-C ≥ 160 mg/dL with stroke was heterogeneous between the subgroups of normal and increased hs-CRP with *I*^*2*^ = 72.9% and a *P*-value of 0.055. The associations of HDL-C < 40 mg/dL, ApoAI < 1.60 g/L, and ApoB < 0.7 g/L with CHD were heterogeneous between the subgroups of normal and increased hs-CRP with *I*^*2*^ > 67% and *P* < 0.1 The relationship of LDL-C ≥ 160 mg/dL with CVD was heterogeneous between subgroups of normal and increased hs-CRP (*I*^*2*^ *=* 84.3%, *P* = 0.011), while the associations of non-HDL-C ≥ 190 mg/dL, and ApoB < 0.7 g/L with CVD showed heterogeneity with *I*^*2*^ of 72.6%, and 72.9% and *P* < 0.1 (Fig. 1).

### Interaction analysis of abnormal lipids levels and hs-CRP on the risk of CVD

We further investigated the potential interactive effect between lipids which had heterogeneous associations with stroke, CHD, and CVD among different hs-CRP subgroups. The results showed significant multiplicative and additive interactions between LDL-C ≥ 160 mg/dL and increased hs-CRP on the risk of stroke [*HR* (95% *CI*): 0.357 (0.154–0.826), *P* = 0.016; RERI (95% CI): -1.397 (-2.690- -0.104), *P* = 0.034)]. Significant multiplicative interactions of HDL-C < 40 mg/dL, ApoAI < 1.60 g/L, and ApoB < 0.70 g/L with increased hs-CRP were also detected for CHD [*HRs* (95% *CIs*): 0.499 (0.259–0.963), 0.552 (0.325–0.935), and 0.509 (0.275–0.942), respectively; all *P * < 0.05]. We also found significant multiplicative and additive interactions of LDL-C ≥ 160 mg/dL and non-HDL-C ≥ 190 mg/dL with increased hs-CRP on the risk of CVD [*HRs* (95% *CIs*): 0.309 (0.153–0.621), and 0.505 (0.295–0.866); RERIs (95% CIs): -1.704 (-3.430-0.021 and − 0.694 (-1.476-0.089), respectively; all *P* < 0.05] (Table 3).

**Table 3:** The interaction analysis of abnormal lipid levels and hs-CRP ≥ 1 mg/L for stroke, CHD, and CVD

Interaction terms	Additive interaction				Multiplicative interaction	
	*RERI* (95% *CI*)	*AP (*95% *CI)*	*P*		*HR* (95% *CI*)	*P*
Stroke						
hs-CRP ≥ 1 mg/L×LDL-C ≥ 160 mg/dL	-1.397 (-2.690- -0.104)	-1.440 (-3.286-0.407)	0.034*		0.357 (0.154–0.826)	0.016*
CHD						
hs-CRP ≥ 1 mg/L×HDL-C < 40 mg/dL	-0.984 (-2.105-0.138)	-0.712 (-1.663-0.237)	0.087		0.499 (0.259–0.963)	0.038*
hs-CRP ≥ 1 mg/L×ApoAI < 1.60 g/L	-0.813 (-1.766-1.140)	-0.454 (-1.007-0.099)	0.094		0.552 (0.325–0.935)	0.027*
hs-CRP ≥ 1 mg/L×ApoB < 0.70 g/L	-0.992 (-2.106-0.122)	-0.627 (-1.478-0.225)	0.081		0.509 (0.275–0.942)	0.031*
CVD						
hs-CRP ≥ 1 mg/L×LDL-C ≥ 160 mg/dL	-1.721 (-2.916- -0.526)	-1.704 (-3.430-0.021)	0.005*		0.309 (0.153–0.621)	0.001*
hs-CRP ≥ 1 mg/L×Non-HDL-C ≥ 190 mg/dL	-0.991 (-1.955- -0.028)	-0.694 (-1.476-0.089)	0.044*		0.505 (0.295–0.866)	0.013*
hs-CRP ≥ 1 mg/L×ApoB < 0.70 g/L	-0.444 (-1.028-0.140)	-0.338 (-0.813-0.138)	0.136		0.691 (0.449–1.065)	0.094

Notes: The model was adjusted for age, gender, smoking, drinking, hypertension, BMI, and diabetes. Hs-CRP: high sensitivity C-reactive Protein, CHD: coronary heart disease, CVD: cardiovascular disease, HDL-C: high-density lipoprotein cholesterol, LDL-C: low-density lipoprotein cholesterol, BMI: body mass index, ApoAI: apolipoprotein AI, ApoB: apolipoprotein B *HR*: hazard ratio, *CI*: confidence intervals, RERI: relative excessive risk interaction, AP: attributable proportion

### Dose-response relationship between lipids indices and CVD by hs-CRP levels

RCS regression indicated that among subjects with normal hs-CRP, ApoB displayed a non-linear pattern with stroke, TC and LDL-C displayed a non-linear pattern with CHD, and ApoB displayed a non-linear pattern with CHD and CVD (all *P* < 0.05). Among subjects with increased hs-CRP, TC, non-HDL-C, and Lp(a) displayed a non-linear pattern with CHD, and TC showed a non-linear pattern with CVD (all *P* < 0.05) (Supplementary Fig. 1 and Table 8).

### Sensitivity analysis

We observed no weakening of the link between hs-CRP and CVD after excluding individuals taking lipid-lowering treatment (n = 19) (Supplementary Table 3). We also increased the cut-off points of hs-CRP and performed sensitivity analyses to explore the validity of our findings. We found that the heterogeneity of the link of non-HDL-C ≥ 190 mg/dL and ApoB < 0.70 g/L with CVD in subgroups of hs-CRP < 1 mg/L and ≥ 1 mg/L still existed when hs-CRP cut-off points were set to 3 mg/L (Supplementary Table 4). The interactions of non-HDL-C ≥ 190 mg/dL with hs-CRP ≥ 3 mg/L on CVD were validated (Supplementary Table 6). The link of ApoB < 0.70 g/L and non-HDL-C ≥ 190 mg/dL with CVD were significantly heterogeneous between the subgroups of hs-CRP < 6 mg/L and ≥ 6 mg/L (Supplementary Table 5), non-HDL-C ≥ 190 mg/dL interacted significantly with hs-CRP ≥ 6 mg/L on the risk of CVD (Supplementary Table 7). Furthermore, all the significant interactions of lipids with the different cut-off points of hs-CRP identified in the sensitivity analysis were negative as in Table 3.

## Discussion

This cohort study suggested a negative interaction between increased hs-CRP and abnormal lipid levels on the risk of CVD. Increased hs-CRP interacted negatively with elevated LDL-C on the risk of stroke, with low HDL-C, ApoAI, and ApoB on the risk of CHD, and with high LDL-C and non-HDL-C on the risk of CVD. This study is the first we know to investigate the combined association of hs-CRP and both conventional and non-conventional lipids on the risk of CVD.

Previous analytic studies reported hs-CRP increase as a significant risk factor for CVD [20, 21, 23]. A cohort study in China indicated that cumulative hs-CRP levels were dose-dependently correlated to cardiovascular events [3]. This study observed that increased hs-CRP contributed to similar hazards of stroke and CHD (HR = 1.25), although the latter association did not reach statistical significance. These results demonstrated the relevance of hs-CRP in the incidence and progression of CVD. The biological process explaining that association might be the following: hs-CRP could directly connect to highly atherogenic oxidized LDL-C and exists within lipid-laden plaques; hs-CRP may contribute to the spread of macrophage in adipose tissue and atherosclerotic lesions by enhancing monocyte adherence and moving into the vascular wall as well as polarizing macrophage M1 [32]. Thus, monitoring hs-CRP levels could be part of CVD prevention measures in the general population.

Remarkably, we also found that increased hs-CRP was a significant predictor of stroke and CVD among subjects with normal lipids levels, but not among subjects with dyslipidemia. Earlier studies investigating the link between hs-CRP and CVD reported various results. In a study from Iran, hs-CRP ≥ 3 mg/L was not a significant predictor of CVD among people with dyslipidemia [20] whereas, in another study, hs-CRP > 3 mg/L contributed significantly to an increased odds of CVD in subjects with HDL-C < 60 mg/dL than subjects with HDL-C ≥ 60 mg/dL [33]. In another study, hs-CRP ≥ 3 mg/L was significantly associated with CHD only among people with LDL-C ≥ 130 mg/dL [34]. In addition, the majority of lipid-lowering medications seem to have anti-inflammatory, antithrombotic, and antihypertensive properties, which were shown to decrease cardiovascular events risk [35]; therefore, we conducted the sensitivity analysis after excluding people taking the lipid-lowering medications at baseline even though they were fewer. The results showed almost no change in the strength of the association between hs-CRP and CVD among subjects with dyslipidemia and normal lipid levels. Moreover, the individuals with detected abnormal lipid levels would have more chances to manage blood lipids during the period of follow-up.

Earlier investigations have suggested that aging is a major factor in the decline of cardiovascular function, which raises the risk of CVD in older persons [36, 37]. Thus, we explored whether age modified the association between increased hs-CRP and CVD. Remarkably, increased hs-CRP strongly contributed to stroke and CVD incidence in subjects aged < 60 years than in subjects aged ≥ 60 years. The stratified analysis by dyslipidemia status showed that the association of increased hs-CRP with stroke and CVD was much stronger among subjects aged < 60 years with normal lipid levels than subjects with dyslipidemia of the same age group, meanwhile, hs-CRP contributed to similar hazards of stroke and CVD among subjects aged ≥ 60 years with dyslipidemia or normal lipid levels; although among seniors the association did not reach statistical significance. These findings suggest a differential effect of dyslipidemia on the association between hs-CRP and CVD among subjects aged < 60 and ≥ 60 years which warrants further investigation.

In our study, high ApoAI was associated with an increased hazard of CVD in the overall study population and among subjects with increased hs-CRP [38, 39]. This result contradicts the previously reported role of ApoAI which has anti-inflammatory features [40] and is associated with a decreased likelihood of CVD in the general population [41]. The association between high ApoAI and CVD found in our study is somewhat akin to the lipid paradox phenomenon. Previous studies have reported a paradox between lipids and CVD prognosis or risk factors. For instance, a previous study reported that higher levels of TC and HDL-C were associated with a decreased risk of arterial fibrillation [42] whereas high LDL-C was related to a decreased hazard of death in another study [38]. However, the biological mechanism explaining the lipids paradox is not elucidated yet; the positive association between high ApoAI and CVD warrants further investigation. RCS analysis showed that among subjects with normal hs-CRP, ApoB displayed a non-linear pattern with CVD while among subjects with increased hs-CRP, TC showed a non-linear pattern with CVD. These results would help to understand the relationship between lipids and CVD with a slight inflammation status of hs-CRP ≥ 1 mg/L.

We further identified negative interactions of increased hs-CRP with high LDL-C on the risk of stroke, low HDL-C, ApoAI, and ApoB on CHD, and higher levels of LDL-C and non-HDL-C on the risk of CVD, suggesting that the combined effects of abnormal lipid levels and increased hs-CRP were smaller than the sum or product of their separate effects [31]. Although studies investigating the interaction between hs-CRP and dyslipidemia on CVD are scarce, our results are different from the one of a previous study which reported a positive interaction between hs-CRP ≥ 3 mg/L with LDL-C ≥ 130 mg/dL on CHD and CVD [43]. Most of the interactions we discovered were further validated with sensitivity analysis using the cut-off points of 3 mg/L and 6 mg/L for hs-CRP showing the validity of our findings regarding the interactive effect of hs-CRP ≥ 1 or ≥ 3 mg/L which indicated a low-grade inflammation [13, 14] and dyslipidemia on CVD. The following hypotheses might explain the biological mechanism involved in that interaction. Mutual antagonism, where increased hs-CRP and abnormal lipid levels individually contribute to CVD occurrence, but when they coexist, they counteract one another’s effect [44]. Another possible hypothesis is the involvement of a fourth factor in the pathway between increased hs-CRP, abnormal lipid levels, and CVD such as the immune system which might damper the joined effect of increased hs-CRP and abnormal lipid levels. Adaptive immune cells may provide a protective reaction at atherosclerotic places, as shown in chronic disorders including CVD by experimental and clinical studies [45, 46]. The immune responses play protective functions in the early and preclinical phases of CVD, but some of them may turn out to be harmful when they can no longer prevent the arterial damage brought on by risk factors in the later stages of atherosclerosis [47]. Repeated measurements of hs-CRP and lipids may better capture the physiological mechanism behind that interaction and provide information about whether their negative interaction continues over time.

### Strengths and limitations

Notably, this study is a twelve-year prospective cohort study that explored the interaction between increased hs-CRP and abnormal lipids levels on CVD incidence, making the findings more credible. To the best of our knowledge, this study is the first to examine the association of lipids on CVD classified according to the levels of hs-CRP and explore the interaction of hs-CRP with both conventional and non-conventional lipids on the risk of CVD on the multiplicative and additive scales. The additive interaction is important to assess rather than only relying exclusively on the multiplicative interaction measures because it is a relevant public health measure [48]. Furthermore, the robustness of the interactions was validated by the sensitivity analysis. However, this study has some limitations. The hs-CRP and lipids were assessed only once, therefore, we could not assess the impact of hs-CRP and lipids changes on the risk of CVD during the follow-up period. Also, the population size was relatively small for interaction analysis and that may limit the statistical power to detect more interactions. Furthermore, there is a significant age variation between the increased hs-CRP and the normal hs-CRP group, so potential confounding bias caused by age might exist even after adjustment for age. The prevalence bias might also exist due to the situation that the individuals with detected abnormal lipids were more likely to manage blood lipids during the period of follow-up. There might be uncontrolled confounding effects caused by unmeasured confounders. In addition, the study involved only one city in China therefore the results could not represent the overall national setting. Finally, the cut-off points of 1 mg/L merely referred to a mild increase of hs-CRP and further exploratory epidemiological studies would be warranted.

## Conclusion

This 12-year prospective cohort study adds to the body of evidence demonstrating the interaction of hs-CRP and dyslipidemia on CVD as well as verifies previous results of increased hs-CRP and dyslipidemia were significant risk factors for CVD in the overall study population. Our findings suggest a negative interaction between hs-CRP and abnormal lipid levels on the risk of CVD. Further large-scale cohort studies with trajectories measurement of lipids and hs-CRP might verify our results as well explore the biological mechanism behind that interaction.

### Electronic supplementary material

Below is the link to the electronic supplementary material.

Supplementary Material 1

## Data Availability

The raw data supporting the conclusions of this article will be made available by the authors, without undue reservation.

## Abbreviations

AP: Attributable proportion
ApoAI: Apolipoprotein AI
ApoB: Apolipoprotein B
BMI: Body mass index
CHD: Coronary heart disease
CI: Confidence intervals
CVD: Cardiovascular disease
DBP: Diastolic blood pressure
DM: Diabetes mellitus
g/L: Grams per liter
HDL-C: High-density lipoprotein cholesterol
HR: Hazard ratio
kg/m^2^: Kilogram per square meter
LDL-C: Low-density lipoprotein
Lp(a): Lipoprotein(a)
mg/dL: Milligrams per deciliter
mg/L: Milligrams per liter
mmHg: Millimeters of mercury
RERI: Relative excessive risk interaction
SBP: Systolic blood pressure
TC: Total cholesterol
TG: Triglycerides

## References

[1] Roth GA, Mensah GA, Johnson CO, Addolorato G, Ammirati E, Baddour LM (2020). Global Burden of Cardiovascular Diseases and Risk factors, 1990–2019: Update from the GBD 2019 study. J Am Coll Cardiol.

[2] Li JJ, Liu HH, Li S (2022). Landscape of cardiometabolic risk factors in chinese population: a narrative review. Cardiovasc Diabetol.

[3] Wang A, Liu J, Li C, Gao J, Li X, Chen S (2017). Cumulative exposure to high-sensitivity C-Reactive protein predicts the risk of Cardiovascular Disease. J Am Heart Assoc.

[4] Holthuis EI, Visseren FLJ, Bots ML, Peters SAE (2021). Risk factor clusters and Cardiovascular Disease in High-Risk Patients: the UCC-SMART study. Glob Heart.

[5] Peters SAE, Wang X, Lam TH, Kim HC, Ho S, Ninomiya T, Knuiman M (2018). Clustering of risk factors and the risk of incident cardiovascular disease in asian and caucasian populations: results from the Asia Pacific Cohort Studies collaboration. BMJ Open.

[6] Frostegård J. Immunity, atherosclerosis and cardiovascular disease. BMC Med. 2013 May;1:11:117. 10.1186/1741-7015-11-117. 10.1186/1741-7015-11-117 PMC3658954 23635324

[7] Jeong SM, Choi S, Kim K, Kim SM, Lee G, Park SY (2018). Effect of change in total cholesterol levels on Cardiovascular Disease among Young adults. J Am Heart Assoc.

[8] Mortensen MB, Nordestgaard BG (2020). Elevated LDL cholesterol and increased risk of myocardial infarction and atherosclerotic cardiovascular disease in individuals aged 70–100 years: a contemporary primary prevention cohort. Lancet.

[9] Kim MK, Han K, Joung HN, Baek KH, Song KH, Kwon HS (2019). Cholesterol levels and development of cardiovascular disease in Koreans with type 2 diabetes mellitus and without pre-existing cardiovascular disease. Cardiovasc Diabetol.

[10] Huang YQ, Huang JY, Liu L, Chen CL, Yu YL, Tang ST (2020). Relationship between triglyceride levels and ischaemic stroke in elderly hypertensive patients. Postgrad Med J.

[11] Park JH, Lee J, Ovbiagele B (2014). Nontraditional serum lipid variables and recurrent stroke risk. Stroke.

[12] Wu H, Wang C, Tuerhongjiang G, Qiao X, Hua Y, She J (2021). Circulating lipid and lipoprotein profiles and their correlation to cardiac function and cardiovascular outcomes in patients with acute myocardial infarction. J Investig Med.

[13] Eklund CM (2009). Proinflammatory cytokines in CRP baseline regulation. Adv Clin Chem.

[14] Musunuru K, Kral BG, Blumenthal RS, Fuster V, Campbell CY, Gluckman TJ (2008). The use of high-sensitivity assays for C-reactive protein in clinical practice. Nat Clin Pract Cardiovasc Med.

[15] Ridker PM, Everett BM, Thuren T, MacFadyen JG, Chang WH, Ballantyne C (2017). Antiinflammatory therapy with Canakinumab for atherosclerotic disease. N Engl J Med.

[16] Nehring SM, Goyal A, Patel BC. C reactive protein. StatPearls Publishing; 2022. 28722873

[17] Tsai MH, Chang CL, Yu YS, Lin TY, Chong CP, Lin YS (2012). Chemical analysis of C-reactive protein synthesized by human aortic endothelial cells under oxidative stress. Anal Chem.

[18] Calabró P, Willerson JT, Yeh ET (2003). Inflammatory cytokines stimulated C-reactive protein production by human coronary artery smooth muscle cells. Circulation.

[19] Dong Y, Wang X, Zhang L, Chen Z, Zheng C, Wang J (2019). High-sensitivity C reactive protein and risk of cardiovascular disease in China-CVD study. J Epidemiol Community Health.

[20] Koosha P, Roohafza H, Sarrafzadegan N, Vakhshoori M, Talaei M, Sheikhbahaei E (2020). High sensitivity C-Reactive protein Predictive Value for Cardiovascular Disease: a nested Case Control from Isfahan Cohort Study (ICS). Glob Heart.

[21] Quispe R, Michos ED, Martin SS, Puri R, Toth PP, Al Suwaidi J (2020). High-sensitivity C-Reactive protein discordance with atherogenic lipid measures and incidence of atherosclerotic Cardiovascular Disease in Primary Prevention: the ARIC Study. J Am Heart Assoc.

[22] Mengozzi M, Kirkham FA, Girdwood EER, Bunting E, Drazich E, Timeyin J (2020). C-Reactive protein predicts further ischemic events in patients with transient ischemic attack or Lacunar Stroke. Front Immunol.

[23] Li ZH, Zhong WF, Lv YB, Kraus VB, Gao X, Chen PL (2019). Associations of plasma high-sensitivity C-reactive protein concentrations with all-cause and cause-specific mortality among middle-aged and elderly individuals. Immun Ageing.

[24] Biasucci LM, CDC/AHA Workshop on Markers of Inflammation and Cardiovascular Disease (2004). Application to clinical and Public Health Practice: clinical use of inflammatory markers in patients with cardiovascular diseases: a background paper. Circulation.

[25] Fonseca FA, Izar MC, High-Sensitivity C-Reactive (2016). Protein and Cardiovascular Disease Across Countries and Ethnicities. Clinics.

[26] Ren Z, Tang W, Fan Y, Shen C, Zhao Y (2018). Association of high sensitive C-reactive protein with stroke: a prospective follow-up study. Chin J Disease Control Prev.

[27] Tang L, Peng H, Xu T, Wang A, Wang G, Tong W (2014). Association of biomarkers of inflammation with dyslipidemia and its components among Mongolians in China. PLoS ONE.

[28] Jin D, Zhu DM, Hu HL, Yao MN, Yin WJ, Tao RX et al. Vitamin D status affects the relationship between lipid profile and high-sensitivity C-reactive protein. Nutr Metab 2020 Jul 14;17:57. 10.1186/s12986-020-00455-x. 10.1186/s12986-020-00455-x PMC7359462 32684941

[29] Joint committee issued Chinese guideline for the management of dyslipidemia in adults (2016). [2016 chinese guideline for the management of dyslipidemia in adults]. Zhonghua xin xue guan bing za zhi.

[30] Dong J, Yang S, Zhuang Q, Sun J, Wei P, Zhao X (2021). The Associations of lipid profiles with Cardiovascular Diseases and Death in a 10-Year prospective cohort study. Front Cardiovasc Med.

[31] Knol MJ, VanderWeele TJ, Groenwold RH, Klungel OH, Rovers MM, Grobbee DE (2011). Estimating measures of interaction on an additive scale for preventive exposures. Eur J Epidemiol.

[32] Yousuf O, Mohanty BD, Martin SS, Joshi PH, Blaha MJ, Nasir K (2013). High-sensitivity C-reactive protein and cardiovascular disease: a resolute belief or an elusive link?. J Am Coll Cardiol.

[33] Bilhorn KR, Luo Y, Lee BT, Wong ND (2012). High-density lipoprotein cholesterol, high-sensitivity C-reactive protein, and cardiovascular disease in United States adults. Am J Cardiol.

[34] Lin GM, Liu K, Colangelo LA, Lakoski SG, Tracy RP (2016). Low-density lipoprotein cholesterol concentrations and association of high-sensitivity C-Reactive protein concentrations with Incident Coronary Heart Disease in the multi-ethnic study of atherosclerosis. Am J Epidemiol.

[35] Tziomalos K, Karagiannis A, Athyros VG (2014). Effects of lipid-lowering agents on inflammation, haemostasis and blood pressure. Curr Pharm Des.

[36] Rodgers JL, Jones J, Bolleddu SI, Vanthenapalli S, Rodgers LE, Shah K, Karia K, Panguluri SK (2019). Cardiovascular Risks Associated with gender and aging. J Cardiovasc Dev Dis.

[37] Yazdanyar A, Newman AB (2009). The burden of cardiovascular disease in the elderly: morbidity, mortality, and costs. Clin Geriatr Med.

[38] Hsu HY, Tsai MC, Yeh TL, Hsu LY, Hwang LC, Chien KL (2021). Association of baseline as well as change in lipid levels with the risk of cardiovascular diseases and all-cause deaths. Sci Rep.

[39] Reddy VS, Bui QT, Jacobs JR, Begelman SM, Miller DP, French WJ (2015). Investigators of National Registry of myocardial infarction (NRMI) 4b–5. Relationship between serum low-density lipoprotein cholesterol and in-hospital mortality following acute myocardial infarction (the lipid paradox). Am J Cardiol.

[40] Umemoto T, Han CY, Mitra P, Averill MM, Tang C, Goodspeed L (2013). Apolipoprotein AI and high-density lipoprotein have anti-inflammatory effects on adipocytes via cholesterol transporters: ATP-binding cassette A-1, ATP-binding cassette G-1, and scavenger receptor B-1. Circ Res.

[41] Holme I, Aastveit AH, Hammar N, Jungner I, Walldius G (2010). Inflammatory markers, lipoprotein components and risk of major cardiovascular events in 65,005 men and women in the apolipoprotein MOrtality RISk study (AMORIS). Atherosclerosis.

[42] Lopez FL, Agarwal SK, Maclehose RF, Soliman EZ, Sharrett AR, Huxley RR (2012). Blood lipid levels, lipid-lowering medications, and the incidence of atrial fibrillation: the atherosclerosis risk in communities study. Circ Arrhythm Electrophysiol.

[43] Nafari A, Mohammadifard N, Haghighatdoost F, Nasirian S, Najafian J, Sadeghi M et al. High-sensitivity C-reactive protein and low-density lipoprotein cholesterol association with incident of cardiovascular events: Isfahan cohort study. BMC Cardiovasc Disord. 2022 May 25;22(1):241. 10.1186/s12872-022-02663-0. 10.1186/s12872-022-02663-0 PMC9131566 35614388

[44] Ould Setti M, Voutilainen A, Tajik B, Niskanen L, Tuomainen TP (2021). Negative interaction of fatty liver and hypertension on cardiovascular mortality in non-diabetic men: 34 years of follow-up. Scand J Gastroenterol.

[45] van der Willik KD, Fani L, Rizopoulos D, Licher S, Fest J, Schagen SB (2019). Balance between innate versus adaptive immune system and the risk of dementia: a population-based cohort study. J Neuroinflammation.

[46] Yamashita T, Sasaki N, Kasahara K, Hirata K (2015). Anti-inflammatory and immune-modulatory therapies for preventing atherosclerotic cardiovascular disease. J Cardiol.

[47] Nilsson J, Hansson GK (2020). Vaccination strategies and Immune Modulation of Atherosclerosis. Circ Res.

[48] VanderWeele TJ, Knol MJ. “A Tutorial on Interaction” Epidemiologic Methods, vol. 3, no. 1, 2014, pp. 33–72. 10.1515/em-2013-0005.

