# Correction: Interactive effect of increased high sensitive C-reactive protein and dyslipidemia on cardiovascular diseases: a 12-year prospective cohort study

**DOI:** 10.1186/s12944-023-01894-0

**Published:** 2023-08-03

**Authors:** Solim Essomandan Clémence Bafei, Xianghai Zhao, Changying Chen, Junxiang Sun, Qian Zhuang, Xiangfeng Lu, Yanchun Chen, Xincheng Gu, Fangyuan Liu, Jialing Mu, Lai Wei, Pengfei Wei, Yunjie Yin, Hankun Xie, Song Yang, Chong Shen

**Affiliations:** 1 Department of Epidemiology, Center for Global Health, School of Public Health, Nanjing Medical University, 211166 Nanjing, China; 2 Department of Cardiology, Affiliated Yixing People’s Hospital of Jiangsu University, People’s Hospital of Yixing City, Yixing, 214200 China; 3 Department of Epidemiology, Fuwai Hospital, National Center for Cardiovascular Diseases, Chinese Academy of Medical Sciences and Peking Union Medical College, Beijing, China; 4 Key Laboratory of Cardiovascular Epidemiology, Chinese Academy of Medical Sciences, Beijing, China; 5 Research Units of Cohort Study on Cardiovascular Diseases and Cancers, Chinese Academy of Medical Sciences, Beijing, China

**Correction: Lipids in Health and Disease 22, 95 (2023)**

10.1186/s12944-023-01836-w

Following publication of the original article [1], the authors would like to correct affiliation 1 into “*Department of Epidemiology, Center for Global Health, School of Public Health, Nanjing Medical University, Nanjing 211166, China*”. Also, an article note is added on page 1 as follows: “Solim Essomandan Clémence Bafei, Xianghai Zhao and Changying Chen contributed equally to this work.”

The original article [1] has been updated.
