# Supplementary material for: Interactive effect of increased high sensitive C-reactive protein and dyslipidemia on cardiovascular diseases: a 12-year prospective cohort study
Source: Lipids Health Dis. 2023 Jul 4;22:95. doi: 10.1186/s12944-023-01836-w (PMC10318784; doi:10.1186/s12944-023-01836-w)
Supplement: Supplementary file 1 — Supplementary Material 1 [file 12944_2023_1836_MOESM1_ESM.docx]

**Supplementary Materials**

**Supplementary Table 1** Cox regression analysis for the association of increased hs-CRP with stroke, CHD, and CVD for the overall population and stratified by age groups and dyslipidemia status.

| Outcomes | Exposure group | Age < 60 years |  |  | Age ≥ 60 years |  | *I^2^*, *P*_Heterogeneity_ |
| --- | --- | --- | --- | --- | --- | --- | --- |
|  |  | *HR* (95% *CI*) | *P* |  | *HR* (95% *CI*) | *P* |  |
| Stroke |  |  |  |  |  |  |  |
|  | Overall |  |  |  |  |  |  |
|  | hs-CRP < 1 mg/L | Ref |  |  | Ref |  | 70.6% |
|  | hs-CRP ≥ 1 mg/L | 2.18 (1.37-3.45) | 0.001* |  | 1.17 (0.93-1.47) | 0.175 | 0.065 |
|  | Dyslipidemia |  |  |  |  |  |  |
|  | Yes |  |  |  |  |  |  |
|  | hs-CRP < 1 mg/L | Ref |  |  | Ref |  |  |
|  | hs-CRP ≥ 1 mg/L | 1.60 (0.85-3.03) | 0.147 |  | 1.20 (0.82-1.75) | 0.352 |  |
|  | No |  |  |  |  |  |  |
|  | hs-CRP < 1 mg/L | Ref |  |  | Ref |  |  |
|  | hs-CRP ≥ 1 mg/L | 3.06 (1.58-5.93) | 0.001* |  | 1.17 (0.88-1.56) | 0.268 |  |
|  | *P*_Heterogeneity_ |  | 0.241 |  |  | 0.919 |  |
| CHD |  |  |  |  |  |  |  |
|  | Overall |  |  |  |  |  |  |
|  | hs-CRP < 1 mg/L | Ref |  |  | Ref |  |  |
|  | hs-CRP ≥ 1 mg/L | 1.21 (0.70-2.08) | 0.499 |  | 1.34 (0.99-1.82) | 0.058 | 0.0% |
|  | Dyslipidemia |  |  |  |  |  | 0.741 |
|  | Yes |  |  |  |  |  |  |
|  | hs-CRP < 1 mg/L | Ref |  |  | Ref |  |  |
|  | hs-CRP ≥ 1 mg/L | 0.96 (0.47-1.98) | 0.917 |  | 1.14 (0.71-1.82) | 0.595 |  |
|  | No |  |  |  |  |  |  |
|  | hs-CRP < 1 mg/L | Ref |  |  | Ref |  |  |
|  | hs-CRP ≥ 1 mg/L | 1.58 (0.70-3.58) | 0.271 |  | 1.48 (1.00-2.21) | 0.052 |  |
|  | *P*_Heterogeneity_ |  | 0.455 |  |  | 0.417 |  |
| CVD |  |  |  |  |  |  |  |
|  | Overall |  |  |  |  |  |  |
|  | hs-CRP < 1 mg/L | Ref |  |  | Ref |  | 12.6% |
|  | hs-CRP ≥ 1 mg/L | 1.67 (1.16-2.39) | 0.006* |  | 1.27 (1.04-1.54) | 0.019* | 0.285 |
|  | Dyslipidemia |  |  |  |  |  |  |
|  | Yes |  |  |  |  |  |  |
|  | hs-CRP < 1 mg/L | Ref |  |  | Ref |  |  |
|  | hs-CRP ≥ 1 mg/L | 1.16 (0.71-1.89) | 0.560 |  | 1.25 (0.91-1.71) | 0.175 |  |
|  | No |  |  |  |  |  |  |
|  |  |  |  |  |  |  |  |
|  | hs-CRP < 1 mg/L | Ref |  |  | Ref |  |  |
|  | hs-CRP ≥ 1 mg/L | 2.50 (1.48-4.22) | 0.001* |  | 1.28 (1.00-1.65) | 0.050* |  |
|  | *P*_Heterogeneity_ |  | 0.076 |  |  | 0.888 |  |

Notes: *: Significance at 0.05, The statistical significance for the heterogeneity test was set at P < 0.1. The model was adjusted for gender, smoking, drinking, hypertension, BMI, and diabetes. BMI: body mass index; CVD: cardiovascular disease, CHD: coronary heart disease, hs-CRP: high-sensitivity C-reactive protein. *HR*: hazard ratio, *CI*: confidence intervals

**Supplementary Table 2** Multiple cox regression analysis of abnormal lipids with the risk of stroke, CHD, and CVD

| Lipids | Groups | Stroke | |  | CHD | |  | CVD | |
| --- | --- | --- | --- | --- | --- | --- | --- | --- | --- |
|  |  | *HR* (95%*CI*) | *P* |  | *HR* (95%CI) | *P* |  | *HR* (95%*CI)* | *P* |
| TC (mg/dL) | < 240 | Ref |  |  | Ref |  |  | Ref |  |
|  | ≥ 240 | 1.14 (0.85-1.54) | 0.390 |  | 1.44 (1.00-2.09) | 0.047* |  | 1.34 (1.06-1.72) | 0.016* |
| TG (mg/dL) | < 200 | ref |  |  | Ref |  |  | Ref |  |
|  | ≥ 200 | 0.98 (0.76-1.26) | 0.878 |  | 0.91 (0.66-1.27) | 0.597 |  | 1.02 (0.83-1.26) | 0.830 |
| LDL-C (mg/dL) | < 160 | Ref |  |  | Ref |  |  |  |  |
|  | ≥ 160 | 1.17 (0.77-1.77) | 0.460 |  | 1.10 (0.63-1.93) | 0.735 |  | 1.24 (0.88-1.76) | 0.214 |
| HDL-C (mg/dL) | 40-60 | Ref |  |  | Ref |  |  | Ref |  |
|  | < 40 | 1.14 (0.86-1.50) | 0.357 |  | 1.33 (0.94-1.87) | 0.103 |  | 1.23 (0.98-1.54) | 0.080 |
|  | ≥ 60 | 1.07 (0.84-1.34) | 0.595 |  | 1.04 (0.77-1.42) | 0.782 |  | 1.11 (0.92-1.36) | 0.282 |
| Non-HDL-C (mg/dL) | < 190 | Ref |  |  | Ref |  |  |  |  |
|  | ≥ 190 | 1.28 (0.93-1.77) | 0.137 |  | 1.26 (0.82-1.95) | 0.287 |  | 1.34 (1.02-1.75) | 0.038* |
| ApoAI (g/dL) | 1.60-2.10 | Ref |  |  | Ref |  |  | Ref |  |
|  | < 1.60 | 1.13 (0.95-1.35) | 0.175 |  | 1.14 (0.95-1.36) | 0.158 |  | 1.14 (0.95-1.36) | 0.152 |
|  | > 2.10 | 1.41 (1.03-1.93) | 0.035* |  | 1.45 (1.05-1.99) | 0.023* |  | 1.44 (1.05-1.97) | 0.025* |
| ApoB (g/dL) | 0.70-0.90 | Ref |  |  | Ref |  |  | Ref |  |
|  | < 0.70 | 1.21 (0.95-1.54) | 0.122 |  | 1.20 (0.94-1.53) | 0.144 |  | 1.21 (0.95-1.54) | 0.130 |
|  | > 0.90 | 1.16 (0.96-1.40) | 0.125 |  | 1.14 (0.94-1.38) | 0.178 |  | 1.15 (0.95-1.390 | 0.150 |
| Lp(a) (mg/dL) | < 90 | Ref |  |  | Ref |  |  | Ref |  |
|  | ≥ 90 | 0.99 (0.84-1.17) | 0.908 |  | 0.99 (0.84-1.17) | 0.907 |  | 0.99 (0.84-1.17) | 0.916 |
| TC/HDL-C | < 3.64 | Ref |  |  | Ref |  |  | Ref |  |
|  | ≥ 3.64 | 0.97 (0.79-1.19) | 0.787 |  | 1.24 (0.96-1.62) | 0.105 |  | 1.09 (0.92-1.29) | 0.327 |
| TG/HDL-C | < 2.27 | Ref |  |  |  |  |  | Ref |  |
|  | ≥ 2.27 | 0.99 (0.80-1.21) | 0.900 |  | 0.93 (0.71-1.21) | 0.593 |  | 0.99 (0.83-1.17) | 0.868 |
| LDL/HDL-C | < 2.02 | Ref |  |  | Ref |  |  | Ref |  |
|  | ≥ 2.02 | 1.07(0.87-1.30) | 0.535 |  | 1.21(0.93-1.57) | 0.160 |  | 1.12 (0.94-1.33) | 0.201 |

Notes: **: Significant at 0.05. The model was adjusted for age, gender, smoking, drinking, hypertension, BMI, and diabetes. TG: triglycerides; TC: total cholesterol, HDL-C: high-density lipoprotein cholesterol, LDL-C: low-density lipoprotein cholesterol, BMI: body mass index, ApoAI: apolipoprotein AI, ApoB: apolipoprotein B, Lp(a): lipoprotein(a), *HR*: hazard ratio, *CI*: confidence intervals**.**

**Supplementary Table 3** Multiple cox regression analysis of increased hs-CRP with stroke, CHD, and CVD among subjects without lipid-lowering treatment

| **Outcome** | **Exposure group** | ***HR* (95%*CI*)** | ***P*** |
| --- | --- | --- | --- |
| Stroke |  |  |  |
|  | Overall |  |  |
|  | hs-CRP < 1 mg/L | Ref |  |
|  | hs-CRP ≥ 1 mg/L | 1.26 (1.03-1.55) | 0.029***** |
|  | Dyslipidemia |  |  |
|  | Yes |  |  |
|  | hs-CRP < 1 mg/L | Ref |  |
|  | hs-CRP ≥ 1 mg/L | 1.20 (0.86- 1.68) | 0.283 |
|  | No |  |  |
|  | hs-CRP < 1 mg/L | Ref |  |
|  | hs-CRP ≥ 1 mg/L | 1.34 (1.03-1.74) | 0.030***** |
| CHD |  |  |  |
|  | Overall |  |  |
|  | hs-CRP < 1 mg/L | Ref |  |
|  | hs-CRP ≥ 1 mg/L | 1.27 (0.97-1.66) | 0.084 |
|  | Dyslipidemia |  |  |
|  | Yes | Ref |  |
|  | hs-CRP < 1 mg/L |  |  |
|  | hs-CRP ≥ 1 mg/L | 1.08 (0.73- 1.62) | 0.700 |
|  | No |  |  |
|  | hs-CRP < 1 mg/L | Ref |  |
|  | hs-CRP ≥ 1 mg/L | 1.41 (0.99-2.02) | 0.058 |
| CVD |  |  |  |
|  | Overall |  |  |
|  | hs-CRP < 1 mg/L | Ref |  |
|  | hs-CRP ≥ 1 mg/L | 1.30 (1.10- 1.55) | 0.003***** |
|  | Dyslipidemia |  |  |
|  | Yes |  |  |
|  | hs-CRP < 1 mg/L | Ref |  |
|  | hs-CRP ≥ 1 mg/L | 1.18 (0.90- 1.55) | 0.231 |
|  | No |  |  |
|  | hs-CRP < 1 mg/L | Ref |  |
|  | hs-CRP ≥ 1 mg/L | 1.42 (1.14- 1.79) | 0.002***** |

Notes: *: Significant at 0.05. The model was adjusted for age, gender, smoking, drinking, hypertension, BMI, and diabetes. Hs-CRP: high sensitivity C-reactive Protein, CHD: coronary heart disease, CVD: cardiovascular disease, *HR*: hazard ratio, *CI*: confidence intervals.

**Supplementary Table 4** Multiple cox regression analysis of abnormal lipids and the risk of stroke, CHD, and CVD stratified by hs-CRP levels < 3 mg/L and ≥ 3 mg/L

| Lipids | Group | hs-CRP < 3 mg/L |  |  | hs-CRP ≥ 3 mg/L |  |  | Heterogeneity test | | |
| --- | --- | --- | --- | --- | --- | --- | --- | --- | --- | --- |
|  |  | *HR* (95% *CI*) | *P* |  | *HR* (95% *CI*) | *P* |  | *I^2^* | *P* | |
| Stroke |  | n = 3611(88.1%) |  |  | n = 487(11.9%) |  |  |  |  | |
| TC (mg/dL) | < 240 | Ref |  |  | Ref |  |  |  |  | |
|  | ≥ 240 | 1.30 (0.94-1.78) | 0.111 |  | 0.62 (0.26-1.47) | 0.276 |  | 69.5% | 0.070 | |
| TG (mg/dL) | < 200 | Ref |  |  | Ref |  |  |  |  | |
|  | ≥ 200 | 0.97 (0.74-1.28) | 0.842 |  | 1.05 (0.59-1.88) | 0.866 |  | 0% | 0.823 | |
| LDL-C (mg/dL) | < 160 | Ref |  |  | Ref |  |  |  |  | |
|  | ≥ 160 | 1.39 (0.90-2.15) | 0.139 |  | 0.45 (0.11-1.87) | 0.272 |  | 65.7% | 0.088 | |
| HDL-C (mg/dL) | 40-60 | Ref |  |  | Ref |  |  |  |  | |
|  | < 40 | 1.12 (0.83-1.51) | 0.473 |  | 1.26 (0.65-2.42) | 0.494 |  | 0% | 0.772 | |
|  | ≥ 60 | 1.01 (0.79-1.30) | 0.930 |  | 1.37 (0.76-2.45) | 0.298 |  | 0% | 0.424 | |
| Non-HDL-C (mg/dL) | < 190 | Ref |  |  | Ref |  |  |  |  | |
|  | ≥ 190 | **1.54 (1.10-2.18)** | **0.012*** |  | 0.47 (0.17-1.33) | 0.155 |  | 85.7% | 0.008***** | |
| ApoAI (g/L) | 1.60-2.10 | Ref |  |  | Ref |  |  |  |  | |
|  | < 1.60 | 1.05 (0.84-1.32) | 0.677 |  | 0.99 (0.59-1.68) | 0.979 |  | 0% | 0.843 | |
|  | > 2.10 | 1.12 (0.73-1.71) | 0.614 |  | 1.79 (0.72-4.45) | 0.207 |  | 0% | 0.496 | |
| ApoB (g/L) | 0.70-0.90 | Ref |  |  | Ref |  |  |  |  | |
|  | < 0.70 | 1.06 (0.76-1.48) | 0.731 |  | 1.16 (0.58-2.31) | 0.681 |  | 0% | 0.834 | |
|  | > 0.90 | **1.38 (1.08-1.76)** | **0.010*** |  | 0.78 (0.44-1.38) | 0.395 |  | 75.7% | 0.043***** | |
| Lp(a) (mg/L) | <90 | Ref |  |  | Ref |  |  |  |  | |
|  | ≥90 | 1.08 (0.87-1.33) | 0.507 |  | 0.61 (0.38-1.00) | 0.052 |  | 82.4% | 0.017***** | |
| TC/HDL-C | <3.64 | Ref |  |  | Ref |  |  |  |  | |
|  | ≥3.64 | 1.05 (0.84-1.31) | 0.672 |  | 0.63 (0.37-1.07) | 0.088 |  | 73.8% | 0.051 | |
| TG/HDL-C | <2.27 | Ref |  |  | Ref |  |  |  |  | |
|  | ≥2.27 | 0.93 (0.74-1.16) | 0.495 |  | 1.39 (0.82-2.37) | 0.226 |  | 20.7% | 0.262 | |
| LDL/HDL-C | <2.02 | Ref |  |  | Ref |  |  |  |  | |
|  | ≥2.02 | 1.17 (0.94-1.46) | 0.161 |  | 0.67 (0.40-1.13) | 0.132 |  | 79.1% | 0.029***** | |
| **CHD** |  | n =3597(88.2%) |  |  | n = 481(11.8%) |  |  |  |  | |
| TC (mg/dL) | < 240 | Ref |  |  | Ref |  |  |  |  | |
|  | ≥240 | 1.40 (0.93-2.09) | 0.105 |  | 1.80 (0.76-4.35) | 0.182 |  | 0% | 0.678 | |
| TG (mg/dL) | < 200 | Ref |  |  | Ref |  |  |  |  | |
|  | ≥200 | 0.91 (0.63-1.31) | 0.606 |  | 0.96 (0.43-2.15) | 0.914 |  | 0% | 0.916 | |
| LDL-C (mg/dL) | <160 | Ref |  |  | Ref |  |  |  |  | |
|  | ≥160 | 1.01 (0.53-1.92) | 0.973 |  | 1.66 (0.49-5.60) | 0.413 |  | 0% | 0 .630 | |
| HDL-C (mg/dL) | 40-60 | Ref |  |  | Ref |  |  |  |  | |
|  | <40 | 1.50 (1.04-2.16) | 0.028***** |  | 0.60 (0.20-1.78) | 0.357 |  | 69.9% | 0.069 | |
|  | ≥60 | 1.00 (0.72-1.40) | 0.996 |  | 1.37 (0.64-2.93) | 0.414 |  | 0% | 0.544 | |
| Non-HDL-C (mg/dL) | <190 | Ref |  |  | Ref |  |  |  |  | |
|  | ≥190 | 1.34 (0.84-2.14) | 0.223 |  | 1.04 (0.36-3.07) | 0.937 |  | 0% | 0.696 | |
| ApoAI (g/L) | 1.60-2.10 | Ref |  |  | Ref |  |  |  |  | |
|  | < 1.60 | 1.35 (1.00-1.83) | 0.005***** |  | 1.32 (0.63-2.78) | 0.457 |  | 0% | 0 .959 | |
|  | > 2.10 | 1.33(0.76-2.31) | 0.306 |  | 2.59 (0.70-9.64) | 0.155 |  | 0% | 0.586 | |
| ApoB (g/L) | 0.70-0.90 | Ref |  |  | Ref |  |  |  |  | |
|  | < 0.70 | 1.73 (1.19-2.52) | 0.004***** |  | 0.60 (0.21-1.67) | 0.325 |  | 80.1% | 0.025***** | |
|  | > 0.90 | 1.13 (0.82-1.57) | 0.454 |  | 0.62 (0.30-1.29) | 0.203 |  | 61.4% | 0.107 | |
| Lp(a) (mg/L) | <90 | Ref |  |  | Ref |  |  |  |  | |
|  | ≥90 | 1.05 (0.80-1.38) | 0.723 |  | 1.54 (0.76-3.11) | 0.228 |  | 0% | 0.427 | |
| TC/HDL-C | <3.64 | Ref |  |  | Ref |  |  |  |  | |
|  | ≥3.64 | 1.28 (0.96-1.70) | 0.091 |  | 1.07 (0.52-2.19) | 0.854 |  | 0% | 0.652 | |
| TG/HDL-C | <2.27 | Ref |  |  | Ref |  |  |  |  | |
|  | ≥2.27 | 0.96 (0.72-1.28) | 0.802 |  | 0.77 (0.38-1.56) | 0.464 |  | 0% | 0.569 | |
| LDL/HDL-C | <2.02 | Ref |  |  | Ref |  |  |  |  | |
|  | ≥2.02 | 1.26 (0.95-1.68) | 0.109 |  | 0.96 (0.48-1.91) | 0.902 |  | 0% | 0.464 | |
| **CVD** |  | n = 3574(88.2%) |  |  | n = 476(11.8) |  |  |  |  | |
| TC (mg/dL) | < 240 | Ref |  |  | Ref |  |  |  |  | |
|  | ≥240 | 1.49 (1.15-1.93) | 0.003***** |  | 0.85 (0.45-1.64) | 0.636 |  | 67.8% | | 0.078 |
| TG (mg/dL) | < 200 | Ref |  |  | Ref |  |  |  | |  |
|  | ≥200 | 1.00 (0.79-1.26) | 0.987 |  | 1.12 (0.69-1.83) | 0.641 |  | 0% | | 0.703 |
| LDL-C (mg/dL) | <160 | Ref |  |  | Ref |  |  |  | |  |
|  | ≥160 | 1.35 (0.93-1.96) | 0.111 |  | 0.86 (0.34-2.17) | 0.767 |  | 0% | | 0.360 |
| HDL-C (mg/dL) | 40-60 | Ref |  |  | Ref |  |  |  | |  |
|  | <40 | 1.28 (0.99-1.64) | 0.058 |  | 1.04 (0.59-1.85) | 0.884 |  | 0% | | 0 .507 |
|  | ≥60 | 1.08 (0.87-1.33) | 0.511 |  | 1.36 (0.82-2.24) | 0.232 |  | 0% | | 0.489 |
| Non-HDL-C (mg/dL) | <190 | Ref |  |  | Ref |  |  |  | |  |
|  | ≥190 | 1.59 (1.19-2.13) | 0.002***** |  | 0.58 (0.26-1.27) | 0.176 |  | 87.9% | | 0.004***** |
| ApoAI (g/L) | 1.60-2.10 | Ref |  |  | Ref |  |  |  | |  |
|  | < 1.60 | 1.15 (0.95-1.40) | 0.159 |  | 1.09 (0.70-1.71) | 0.712 |  | 0% | | 0.832 |
|  | > 2.10 | 1.28(0.90-1.82) | 0.168 |  | 2.97(1.38-6.41) | 0.006***** |  | 40.4% | | 0.195 |
| ApoB (g/L) | 0.70-0.90 | Ref |  |  | Ref |  |  |  | |  |
|  | < 0.70 | 1.31(1.00-1.72) | 0.048***** |  | 0.81(0.44-1.47) | 0.480 |  | 58.9% | | 0.119 |
|  | > 0.90 | 1.30 (1.06-1.61) | 0.012***** |  | 0.65 (0.41-1.04) | 0.072 |  | 89.2% | | 0.002***** |
| Lp(a) (mg/L) | <90 | Ref |  |  | Ref |  |  |  | |  |
|  | ≥90 | 1.03 (0.86-1.23) | 0.753 |  | 0.79 (0.52-1.19) | 0.259 |  | 33.8% | | 0.219 |
| TC/HDL-C | <3.64 | Ref |  |  | Ref |  |  |  | |  |
|  | ≥3.64 | 1.17 (0.97-1.42) | 0.092 |  | 0.72 (0.46-1.12) | 0.140 |  | 79.5% | | 0.027***** |
| TG/HDL-C | <2.27 | Ref |  |  | Ref |  |  |  | |  |
|  | ≥2.27 | 0.96 (0.80-1.16) | 0.706 |  | 1.11 (0.71-1.73) | 0.648 |  | 0% | | 0.580 |
| LDL/HDL-C | <2.02 | Ref |  |  | Ref |  |  |  | |  |
|  | ≥2.02 | 1.22 (1.01-1.47) | 0.035***** |  | 0.73 (0.47-1.12) | 0.151 |  | 82.8% | | 0.016***** |

Notes: *: Significance at 0.05. The statistical significance for the heterogeneity test was set at *P* < 0.1. The model was adjusted for age, gender, smoking, drinking, hypertension, BMI, and diabetes. Hs-CRP: high sensitivity C-reactive Protein, CHD: coronary heart disease, CVD: cardiovascular disease, TC: total cholesterol, HDL-C: high-density lipoprotein cholesterol, LDL-C: low-density lipoprotein cholesterol, BMI: body mass index, ApoAI: apolipoprotein AI, ApoB: apolipoprotein B, Lp(a): lipoprotein(a), *HR*: hazard ratio, *CI*: confidence intervals.

**Supplementary Table 5** Multiple cox regression analysis of abnormal lipids indices and the risk of stroke, CHD, and CVD stratified by hs-CRP levels < 6 mg/L and ≥ 6 mg/L

| Lipids | Groups | hs-CRP < 6 mg/L |  |  | hs-CRP ≥ 6 mg/L |  |  | Heterogeneity test | | |  |
| --- | --- | --- | --- | --- | --- | --- | --- | --- | --- | --- | --- |
|  |  | *HR* (95%*CI*) | *P* |  | *HR* (95%*CI*) | *P* |  | *I^2^* | *P* | |  |
| Stroke |  | *n* = 3916(95.6%) |  |  | *n* = 182(4.4%) |  |  |  | |  |  |
| TC (mg/dL) | < 240 | Ref |  |  | Ref |  |  |  | |  |  |
|  | ≥240 | 1.20 (0.88-1.62) | 0.249 |  | 0.43 (0.09-2.04) | 0.289 |  | 52.3% | | 0.148 |  |
| TG (mg/dL) | < 200 | Ref |  |  | Ref |  |  |  | |  |  |
|  | ≥200 | 0.95 (0.74-1.24) | 0.717 |  | 1.47 (0.55-3.91) | 0.443 |  | 0% | | 0.548 |  |
| LDL-C (mg/dL) | <160 | Ref |  |  | Ref |  |  |  | |  |  |
|  | ≥160 | 1.25 (0.83-1.90) | 0.292 |  | 0.00(0.00-NA) | 0.991 |  | NA | | NA |  |
| HDL-C (mg/dL) | 40-60 | Ref |  |  | Ref |  |  |  | |  |  |
|  | <40 | 1.08 (0.82-1.44) | 0.582 |  | 2.73 (0.81-9.16) | 0.105 |  | 0% | | 0.440 |  |
|  | ≥60 | 1.02 (0.81-1.30) | 0.848 |  | 2.30 (0.89-5.93) | 0.084 |  | 0% | | 0.322 |  |
| Non-HDL-C (mg/dL) | <190 | Ref |  |  | Ref |  |  |  | |  |  |
|  | ≥190 | 1.35 (0.97-1.87) | 0.074 |  | 0.34 (0.05-2.63) | 0.302 |  | 52.4% | | 0.147 |  |
| ApoAI (g/L) | 1.60-2.10 | Ref |  |  | Ref |  |  |  | |  |  |
|  | < 1.60 | 1.06 (0.85-1.31) | 0.603 |  | 0.60 (0.23-1.55) | 0.291 |  | 39.9% | | 0.197 |  |
|  | > 2.10 | 1.14 (0.76-1.71) | 0.537 |  | 1.62 (0.49-5.35) | 0.427 |  | 0% | | 0.704 |  |
| ApoB (g/L) | 0.70-0.90 | Ref |  |  | Ref |  |  |  | |  |  |
|  | < 0.70 | 1.10 (0.81-1.51) | 0.533 |  | 0.81 (0.26-2.51) | 0.713 |  | 0% | | 0.704 |  |
|  | > 0.90 | 1.31 (1.04-1.65) | 0.021* |  | 0.47 (0.17-1.28) | 0.139 |  | 85.2% | | 0.009***** |  |
| LP(a) (mg/L) | <90 | Ref |  |  | Ref |  |  |  | |  |  |
|  | ≥90 | 1.01 (0.82-1.23) | 0.958 |  | 0.66 (0.29-1.53) | 0.334 |  | 9.4% | | 0.293 |  |
|  | ≥3.64 | 0.98 (0.80-1.21) | 0.854 |  | 0.55 (0.20-1.51) | 0.553 |  | 33.7% | | 0.219 |  |
| TG/HDL-C | <2.27 | Ref |  |  | Ref |  |  |  | |  |  |
|  | ≥2.27 | 0.96 (0.78-1.18) | 0.686 |  | 1.40 (0.53-3.73) | 0.501 |  | 0% | | 0.593 |  |
| LDL/HDL-C | <2.02 | Ref |  |  | Ref |  |  |  | |  |  |
|  | ≥2.02 | 1.12 (0.91-1.38) | 0.285 |  | 0.47 (0.18-1.20) | 0.115 |  | 80.6% | | 0.023***** |  |
| **CHD** |  | n = 3899(95.6%) |  |  | n = 179(4.4%) |  |  |  | |  |  |
| TC (mg/dL) | < 240 | Ref |  |  | Ref |  |  |  | |  |  |
|  | ≥240 | 1.48 (1.02-2.15) | 0.042***** |  | 1.60 (0.32-8.09) | 0.571 |  | 0% | | 0.952 |  |
| TG (mg/dL) | < 200 | Ref |  |  | Ref |  |  |  | |  |  |
|  | ≥200 | 0.91 (0.64-1.28) | 0.568 |  | 1.05 (0.28-3.92) | 0.940 |  | 0% | | 0.882 |  |
| LDL-C (mg/dL) | <160 | Ref |  |  | Ref |  |  |  | |  |  |
|  | ≥160 | 1.19 (0.68-2.09) | 0.544 |  | 0.00 (0.00-NA) | 0.993 |  | NA | | NA |  |
| HDL-C (mg/dL) | 40-60 | Ref |  |  | Ref |  |  |  | |  |  |
|  | <40 | 1.29 (0.91-1.84) | 0.157 |  | 2.56 (0.62-10.65) | 0.196 |  | 0% | | 0.621 |  |
|  | ≥60 | 1.00 (0.73-1.38) | 0.984 |  | 1.91 (0.63-5.76) | 0.253 |  | 0% | | 0.490 |  |
| Non-HDL-C (mg/dL) | <190 | Ref |  |  | Ref |  |  |  | |  |  |
|  | ≥190 | 1.38 (0.90-2.13) | 0.144 |  | 0.00 (0.00-NA) | 0.994 |  | NA | | NA |  |
| ApoAI (g/L) | 1.60-2.10 | Ref |  |  | Ref |  |  |  | |  |  |
|  | < 1.60 | 1.34 (1.01-1.80) | 0.041***** |  | 1.49 (0.44-5.04) | 0.521 |  | 0% | | 0.900 |  |
|  | > 2.10 | 1.27 (0.74-2.19) | 0.387 |  | 3.93 (0.80-19.30) | 0.092 |  | 0% | | 0.574 |  |
| ApoB (g/L) | 0.70-0.90 | Ref |  |  | Ref |  |  |  | |  |  |
|  | < 0.70 | 1.69 (1.17-2.42)* | 0.005***** |  | 0.30 (0.06-1.47) | 0.137 |  | 88.0% | | 0.004***** |  |
|  | > 0.90 | 1.09 (0.80-1.48) | 0.582 |  | 0.51 (0.17-1.57) | 0.241 |  | 53.1% | | 0 .144 |  |
| Lp(a) (mg/L) | <90 | Ref |  |  | Ref |  |  |  | |  |  |
|  | ≥90 | 1.09 (0.84-1.42) | 0.524 |  | 1.53 (0.53-4.47) | 0.434 |  | 0% | | 0.665 |  |
| TC/HDL-C | <3.64 | Ref |  |  | Ref |  |  |  | |  |  |
|  | ≥3.64 | 1.25 (0.96-1.65) | 0.104 |  | 1.30 (0.44-3.81) | 0.636 |  | 0% | | 0.955 |  |
| TG/HDL-C | <2.27 | Ref |  |  | Ref |  |  |  | |  |  |
|  | ≥2.27 | 0.95 (0.72-1.25) | 0.702 |  | 0.73 (0.24-2.24) | 0.585 |  | 0% | | 0.677 |  |
| LDL/HDL-C | <2.02 | Ref |  |  | Ref |  |  |  | |  |  |
|  | ≥2.02 | 1.21 (0.92-1.59) | 0.166 |  | 1.14 (0.39-3.28) | 0.816 |  | 0% | | 0.926 |  |
| **CVD** |  | n = 3872(95.6%) |  |  | n = 178(4.4%) |  |  |  | |  |  |
| TC (mg/dL) | < 240 | Ref |  |  | Ref |  |  |  | |  |  |
|  | ≥240 | 1.41 (1.10-1.81)* | 0.007***** |  | 0.70 (0.23-2.18) | 0.541 |  | 44.4% | | 0.180 |  |
| TG (mg/dL) | < 200 | Ref |  |  | Ref |  |  |  | |  | |
|  | ≥200 | 1.00 (0.80-1.24) | 0.967 |  | 1.50 (0.68-3.31) | 0.319 |  | 0% | | 0 .462 | |
| LDL-C (mg/dL) | <160 | Ref |  |  | Ref |  |  |  | |  | |
|  | ≥160 | 1.36 (0.97-1.93) | 0.079 |  | 0.00 (0.00-NA) | 0.988 |  | NA | | NA | |
| HDL-C (mg/dL) | 40-60 | Ref |  |  | Ref |  |  |  | |  | |
|  | <40 | 1.18 (0.93-1.50) | 0.166 |  | 2.47 (0.96-6.36) | 0.061 |  | 0% | | 0.352 | |
|  | ≥60 | 1.06 (0.86-1.30) | 0.584 |  | 2.58 (1.22-5.45) | 0.013* |  | 49.1% | | 0.161 | |
| Non-HDL-C (mg/dL) | <190 | Ref |  |  | Ref |  |  |  | |  | |
|  | ≥190 | 1.45 (1.10-1.92) | 0.008***** |  | 0.20 (0.03-1.54) | 0.123 |  | 87.7% | | 0.004***** | |
| ApoAI (g/L) | 1.60-2.10 | Ref |  |  | Ref |  |  |  | |  | |
|  | < 1.60 | 1.15 (0.96-1.39) | 0.129 |  | 0.84 (0.40-1.79) | 0.658 |  | 0% | | 0.404 | |
|  | > 2.10 | 1.27 (0.90-1.79) | 0.168 |  | 3.68 (1.37-9.87) | 0.010***** |  | 18.2% | | 0.269 | |
| ApoB (g/L) | 0.70-0.90 | Ref |  |  | Ref |  |  |  | |  | |
|  | < 0.70 | 1.28 (0.99-1.65) | 0.058 |  | 0.56(0.23-1.37) | 0.200 |  | 78.2% | | 0.032***** | |
|  | > 0.90 | 1.23(1.01-1.50) | 0.040***** |  | 0.42(0.19-0.94) | 0.034* |  | 92.0% | | < 0.001***** | |
| Lp(a) (mg/L) | <90 | Ref |  |  | Ref |  |  |  | |  | |
|  | ≥90 | 1.00 (0.84-1.18) | 0.965 |  | 0.89 (0.45-1.74) | 0.728 |  | 0% | | 0.747 | |
|  | ≥3.64 | 1.11 (0.93-1.32) | 0.271 |  | 0.73 (0.34-1.57) | 0.422 |  | 25.0% | | 0.248 | |
| TG/HDL-C | <2.27 | Ref |  |  | Ref |  |  |  | |  | |
|  | ≥2.27 | 0.98 (0.82-1.17) | 0.818 |  | 1.05 (0.49-2.22) | 0.905 |  | 0% | | 0.876 | |
| LDL/HDL-C | <2.02 | Ref |  |  | Ref |  |  |  | |  | |
|  | ≥2.02 | 1.17 (0.98-1.39) | 0.089 |  | 0.61 (0.30-1.25) | 0.177 |  | 77.8% | | 0.034* | |

Notes: *: Significance at 0.05. The statistical significance for heterogeneity test was set at *P* < 0.1. The model was adjusted for age, gender, smoking, drinking, hypertension, BMI, and diabetes. Hs-CRP: high sensitivity C-reactive Protein, CHD: coronary heart disease, CVD: cardiovascular disease, TC: total cholesterol, HDL-C: high-density lipoprotein cholesterol, LDL-C: low-density lipoprotein cholesterol, BMI: body mass index, ApoAI: apolipoprotein AI, ApoB: apolipoprotein B, Lp(a): lipoprotein(a), *HR*: hazard ratio, *CI*: confidence intervals.

**Supplementary Table 6** Interaction analysis of abnormal lipid levels and hs-CRP ≥ 3 mg/L for stroke, CHD, and CVD

| Interactive terms | Additive interaction | | |  | Multiplicative interaction | |
| --- | --- | --- | --- | --- | --- | --- |
|  | *RERI* (95% *CI*) | *AP (*95% *CI)* | *P* |  | *HR* (95% *CI*) | *P* |
| Stroke |  |  |  |  |  |  |
| hs-CRP ≥ 3 mg/L×TC ≥ 240 mg/L | -0.776 (-1.471- -0.081) | -1.316 (-3.278-0.646) | 0.029* |  | 0.426 (0.174-1.046) | 0.063 |
| hs-CRP ≥ 3 mg/L×LDL-C ≥ 160 mg/dL | -0.995 (-1.884- -0.105) | -2.386 (-7.233-2.461) | 0.028* |  | 0.294 (0.067-1.285) | 0.104 |
| hs-CRP ≥ 3 mg/L×TC/HDL-C ≥ 3.64 | -0.511 (-1.082-0.059) | -0.652 (-1.381-0.077) | 0.079 |  | 0.599 (0.353-1.019) | 0.059 |
| hs-CRP ≥ 3 mg/L×Non-HDL-C ≥ 190 mg/dL | -1.196 (-1.967- -0.425) | -2.579 (-6.244-1.085) | 0.002* |  | 0.272 (0.094-0.788) | 0.016* |
| hs-CRP ≥ 3 mg/L×LDL/HDL-C ≥ 2.02 | -0.674 (-1.289- -0.059) | -0.810 (-1.611-0.009) | 0.032* |  | 0.532 (0.313-0.903) | 0.020* |
| hs-CRP ≥ 3 mg/L×ApoB ≥ 0.90 g/L | -0.758 (-1.402- -0.114) | -0.827 (-1.637- -0.016) | 0.021* |  | 0.513 (0.302-0.871) | 0.014* |
| hs-CRP ≥ 3 mg/L×Lp(a) ≥ 90 mg/L | -0.544 (-1.128-0.041) | -0.679 (-1.411-0.054) | 0.068 |  | 0.587 (0.346-0.996) | 0.048* |
| **CHD** |  |  |  |  |  |  |
| hs-CRP ≥ 3 mg/L×HDL-C < 40 mg/dL | -0.953 (-1.848- -0.058) | -1.561 (-4.214- 1.092) | 0.037* |  | 0.383 (0.128-1.145) | 0.086 |
| hs-CRP ≥ 3 mg/L×ApoB < 0.70 g/L | -0.749 (-1.747-0.249) | -0.857 (-2.590-0.877) | 0.141 |  | 0.533 (0.196-1.447) | 0.217 |
| **CVD** |  |  |  |  |  |  |
| hs-CRP ≥ 3 mg/L×TC ≥ 240 mg/L | -0.746 (-1.444- -0.047) | -0.85 (-2.023- -0.330) | 0.036* |  | 0.522 (0.264-1.031) | 0.061 |
| hs-CRP ≥ 3 mg/L×Non-HDL-C ≥ 190 mg/dL | -1.153 (-1.861- -0.445) | -1.872 (-4.121- 0.378) | 0.001* |  | 0.331 (0.145-0.752) | 0.008* |
| hs-CRP ≥ 3 mg/L×TC/HDL-C ≥ 3.64 | -0.574 (-1.114- -0.033) | -0.601 (-1.195- -0.006) | 0.037* |  | 0.600 (0.384-0.939) | 0.025* |
| hs-CRP ≥ 3 mg/L×LDL/HDL-C ≥ 2.02 | -0.694 (-1.253- -0.134) | -0.727 (-1.372- -0.082) | 0.015* |  | 0.547 (0.350-0.855) | 0.008* |
| hs-CRP ≥ 3 mg/L×ApoB ≥ 0.90 g/L | -0.672 (-1.212- -0.132) | -0.744 (-1.401- -0.086) | 0.015* |  | 0.548 (0.350-0.858) | 0.009* |

Notes: *: Significance at 0.05. The model was adjusted for age, gender, smoking, drinking, hypertension, BMI, and diabetes. Hs-CRP: high sensitivity C-reactive Protein, CHD: coronary heart disease, CVD: cardiovascular disease, TC: total cholesterol, HDL-C: high-density lipoprotein cholesterol, LDL-C: low-density lipoprotein cholesterol, BMI: body mass index, ApoAI: apolipoprotein AI, ApoB: apolipoprotein B, Lp(a): lipoprotein(a), *HR*: hazard ratio, *CI*: confidence intervals, RERI: relative excessive risk interaction, AP: attributable proportion.

**Supplementary Table 7** Interaction analysis of abnormal lipid levels and hs-CRP ≥ 6 mg/L for stroke, CHD, and CVD

| Interactive terms | Additive interaction | | |  | Multiplicative interaction | |
| --- | --- | --- | --- | --- | --- | --- |
|  | *RERI* (95% *CI*) | *AP (*95% *CI)* | *P* |  | *HR* (95% *CI*) | *P* |
| Stroke |  |  |  |  |  |  |
| hs-CRP ≥ 6 mg/L×LDL/HDL-C ≥ 2.02 | -0.715 (-1.500-0.071) | -1.221 (-2.964-0.522) | 0.074 |  | 0.443 (0.183-1.072) | 0.071 |
| hs-CRP ≥ 6 mg/L×ApoB ≥ 0.90 g/L | -0.722 (-1.546-0.102) | -1.034 (-2.569-0.502) | 0.086 |  | 0.478 (0.201-1.134) | 0.094 |
| CHD |  |  |  |  |  |  |
| hs-CRP ≥ 6 mg/L×ApoB < 0.7 g/L | -1.381 (-2.627- -0.136) | -2.294 (-7.078- 2.490) | 0.030* |  | 0.272 (0.060-1.239) | 0.092 |
| CVD |  |  |  |  |  |  |
| hs-CRP ≥ 6 mg/L×Non-HDL-C ≥ 190 mg/dL | -1.388 (-2.187- -0.589) | -5.047 (-17.042-6.948) | <0.001* |  | 0.156 (0.021-1.162) | 0.070 |
| hs-CRP ≥ 6 mg/L×LDL/HDL-C ≥ 2.02 | -0.665(-1.462- 0.131) | -0.726 (-1.764-0.311) | 0.102 |  | 0.556 (0.281-1.098) | 0.091 |
| hs-CRP ≥ 6 mg/L×ApoB < 0.70 g/L | -0.321 (-1.150- 0.508) | -0.336 (-1.339- 0.668) | 0.448 |  | 0.738 (0.326-1.671) | 0.466 |
| hs-CRP ≥ 6 mg/L×ApoB ≥ 0.90 g/L | -0.730 (-1.511- 0.051) | -0.872 (-2.023- 0.280) | 0.067 |  | 0.516 (0.259-1.029) | 0.060 |

Notes: *: Significance at 0.05. The model was adjusted for age, gender, smoking, drinking, hypertension, BMI, and diabetes. Hs-CRP: high sensitivity C-reactive Protein, CHD: coronary heart disease, CVD: cardiovascular disease, TC: total cholesterol, HDL-C: high-density lipoprotein cholesterol, LDL-C: low-density lipoprotein cholesterol, BMI: body mass index, ApoAI: apolipoprotein AI, ApoB: apolipoprotein B, *HR*: hazard ratio, *CI*: confidence intervals, RERI: relative excessive risk interaction, AP: attributable proportion.


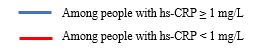


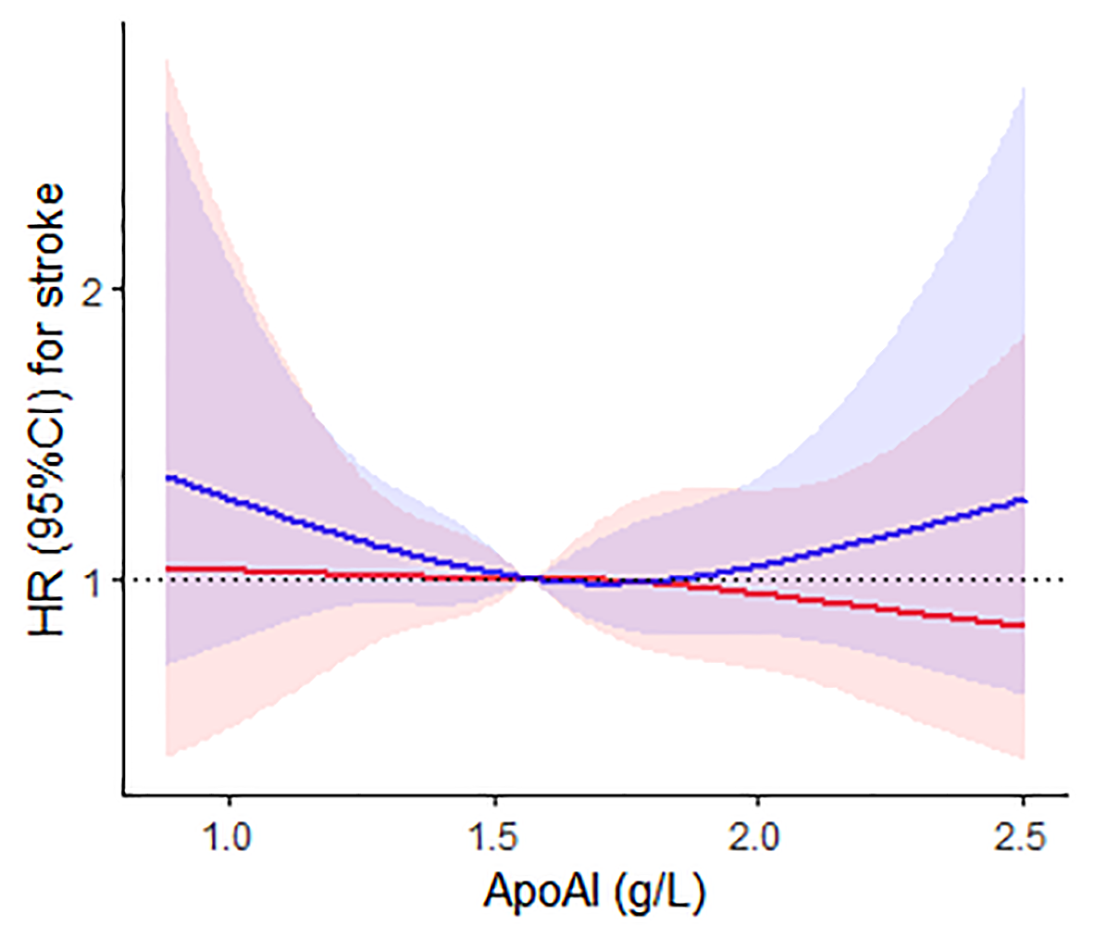

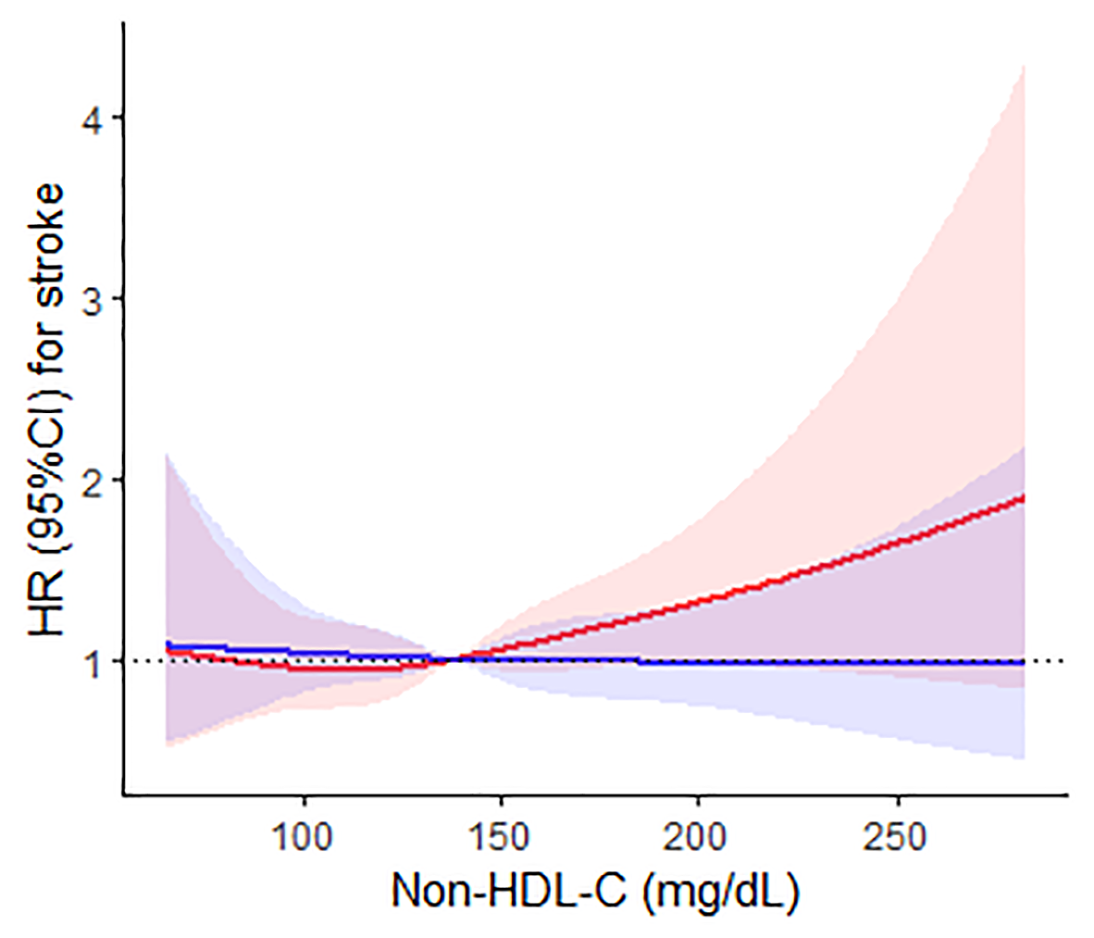

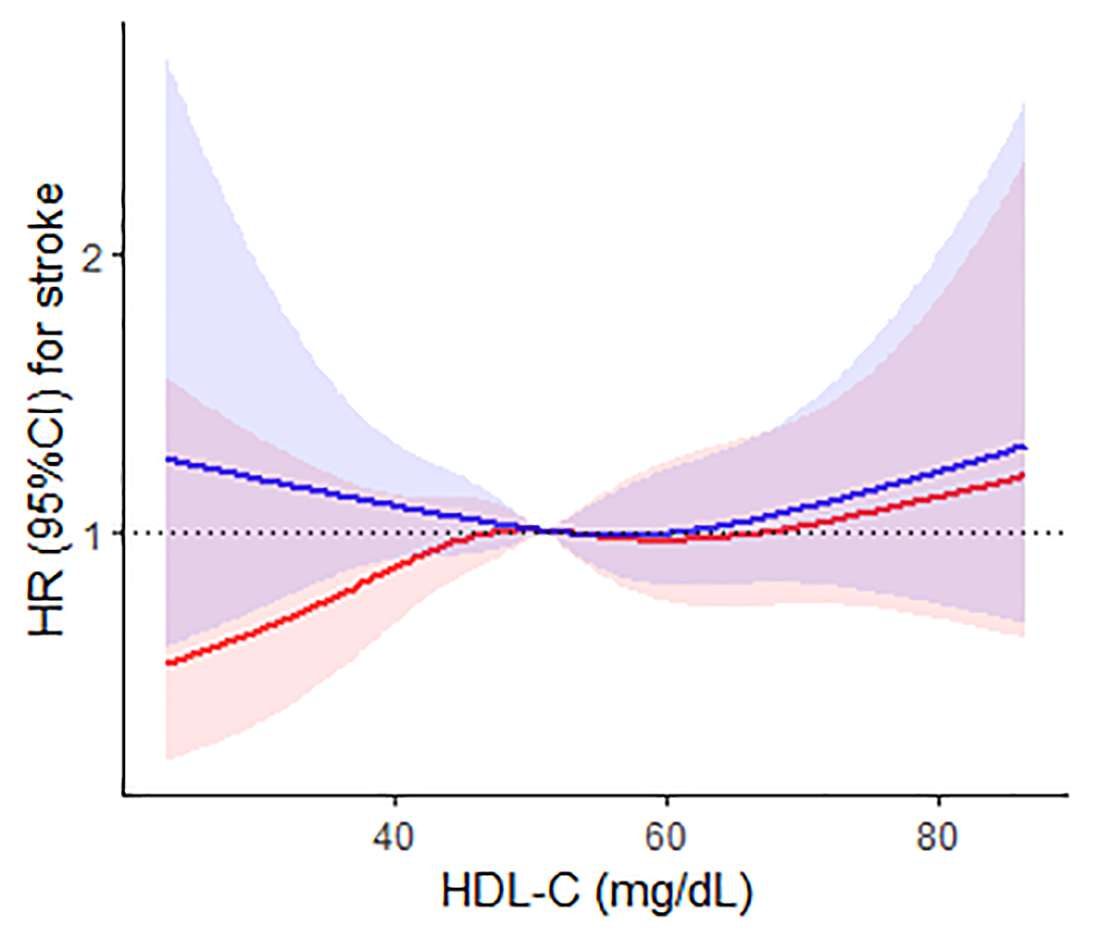

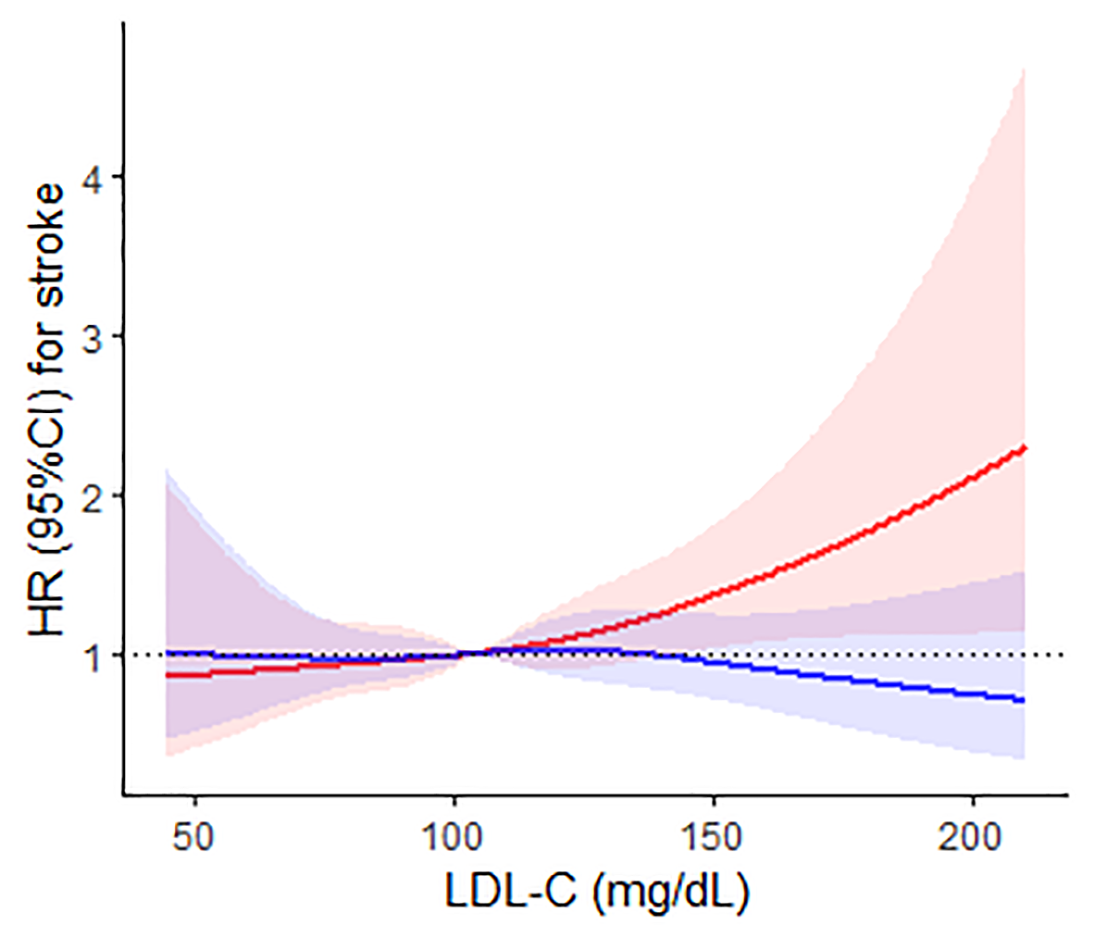

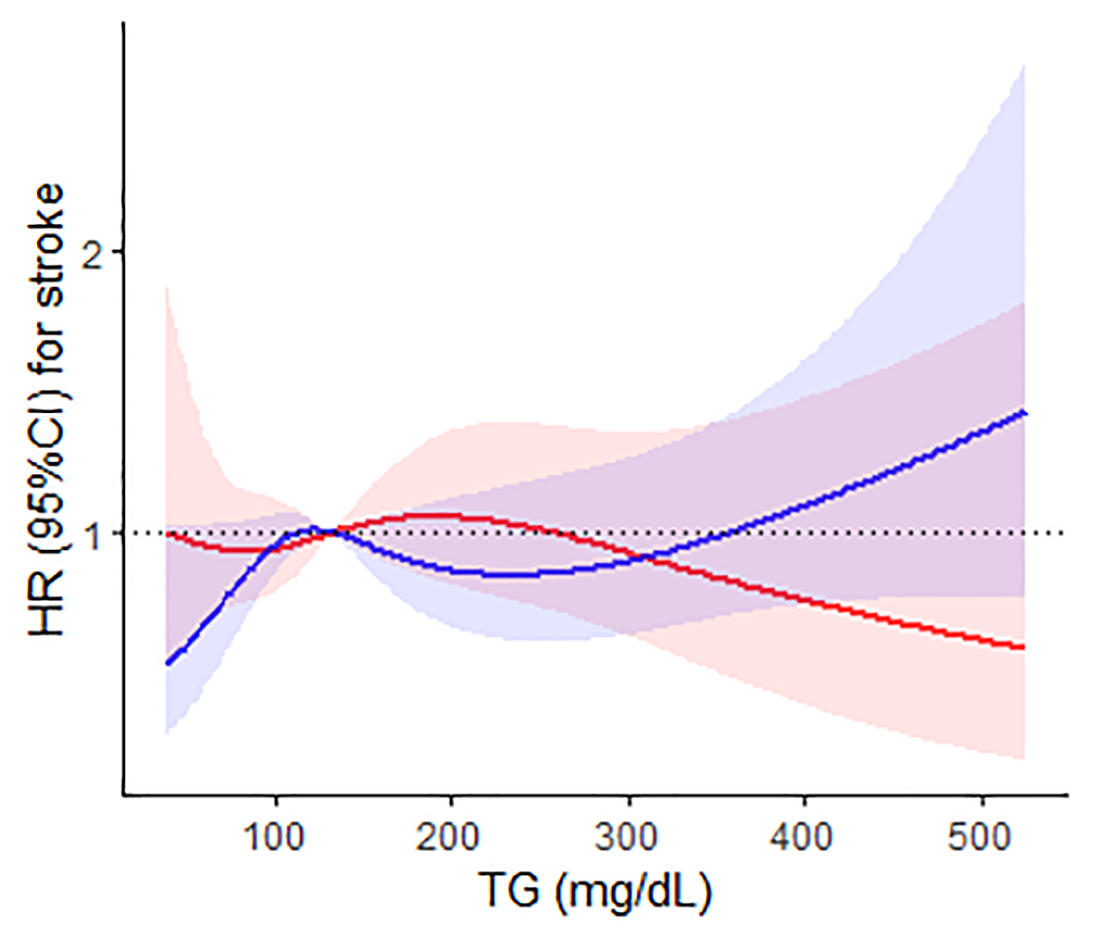

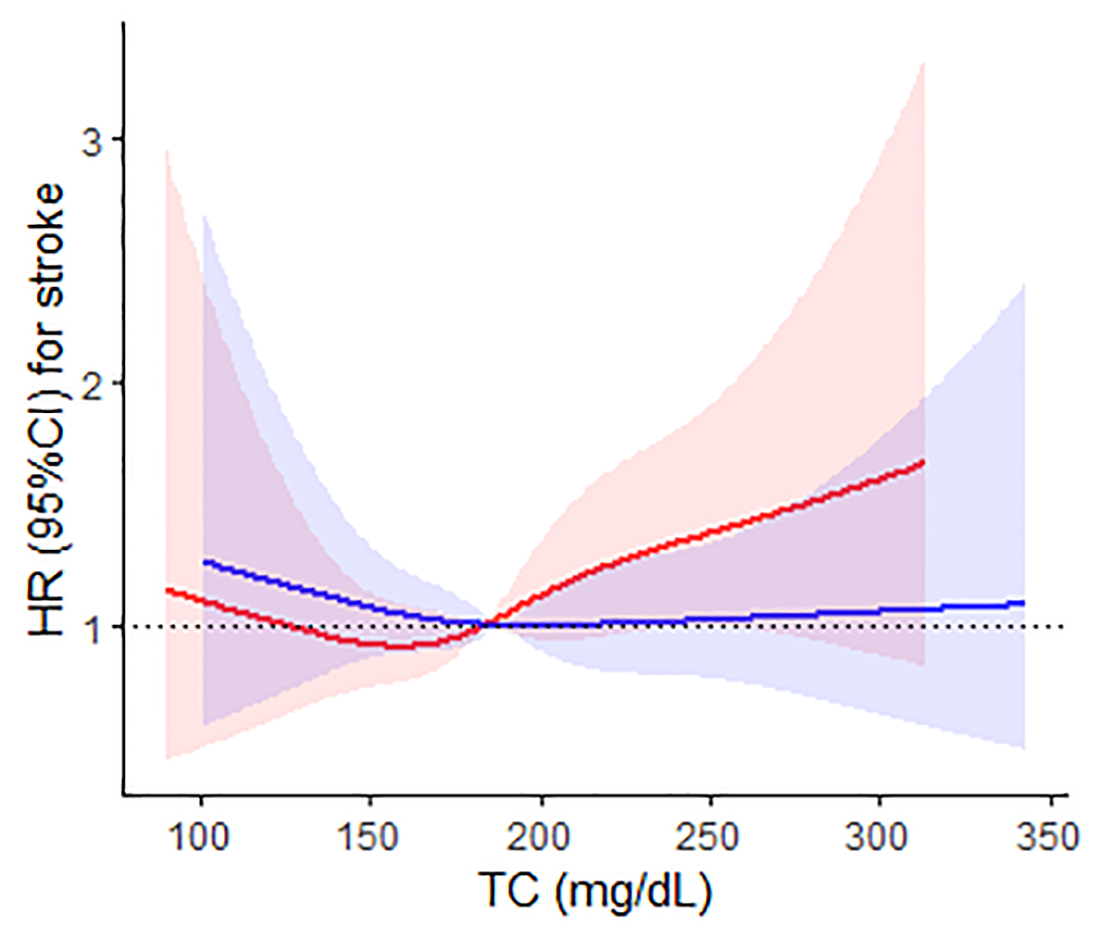


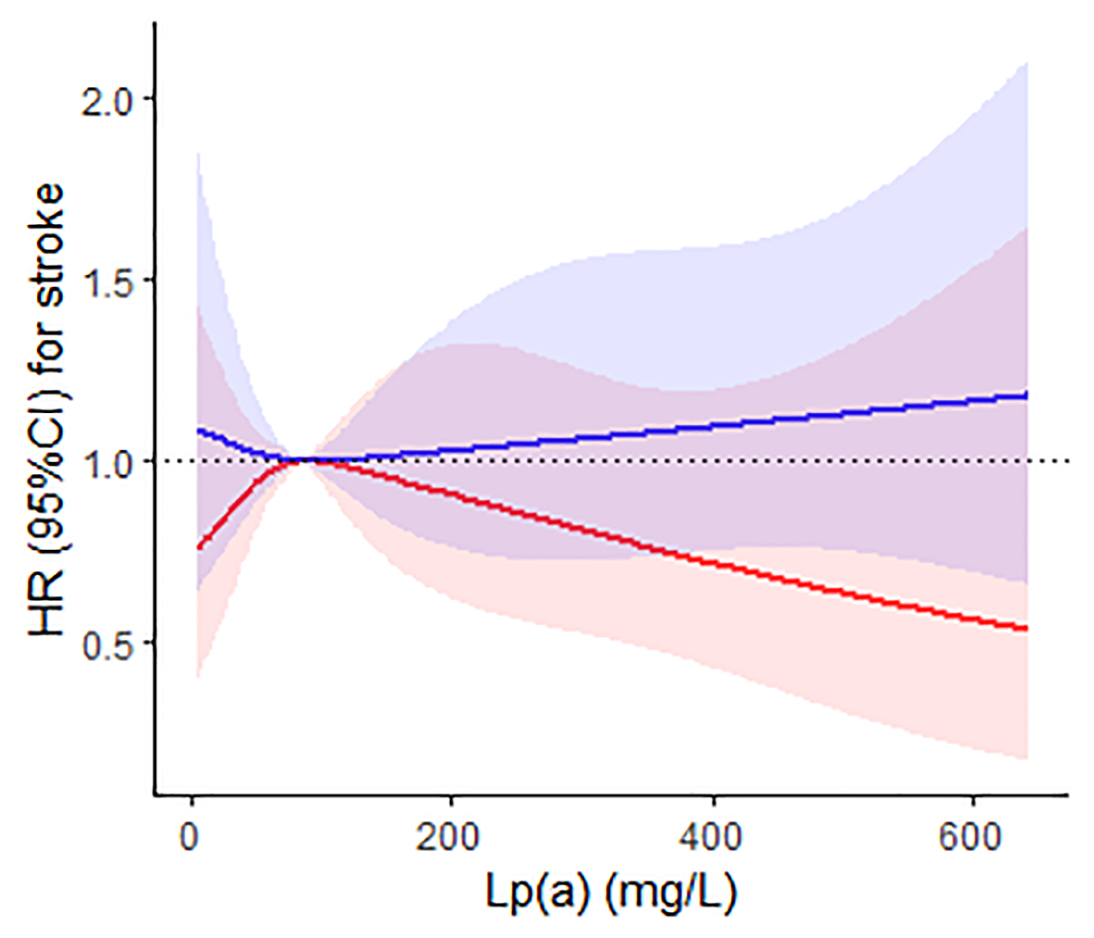

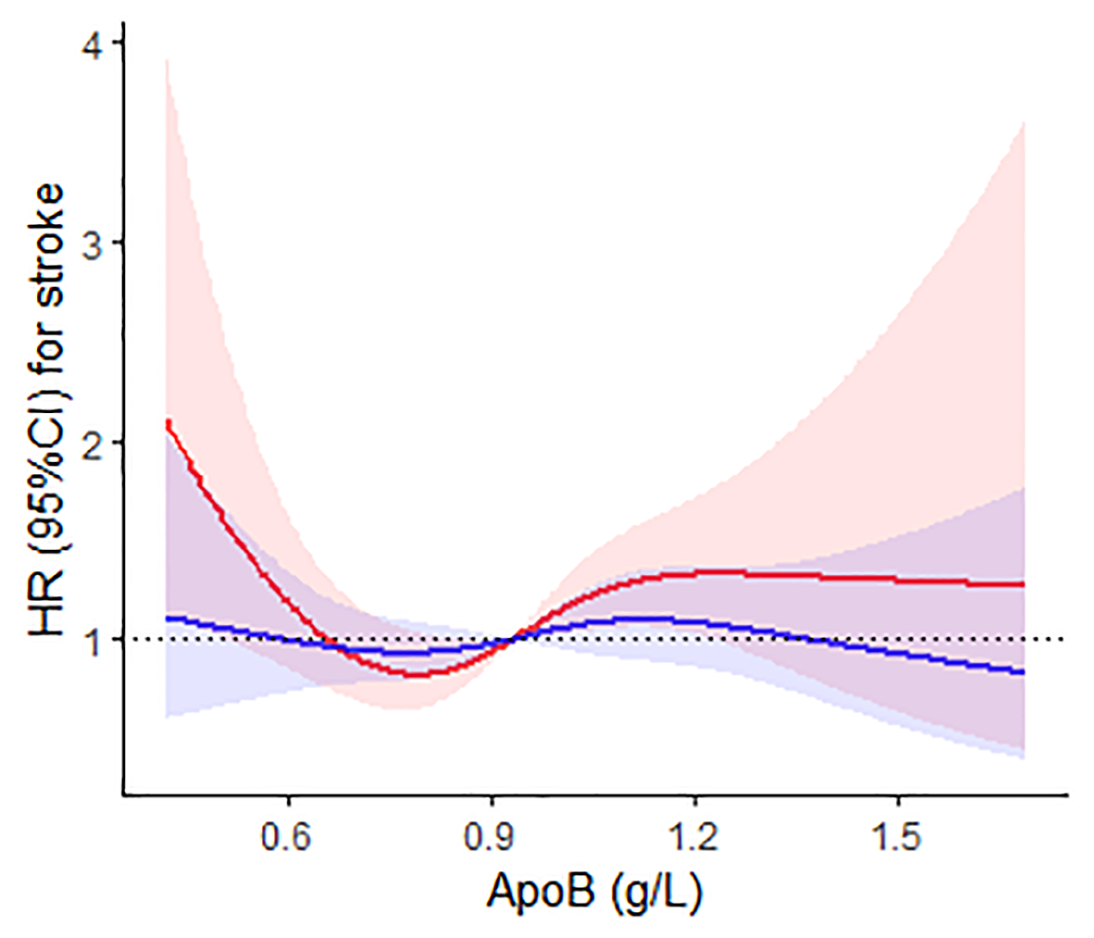

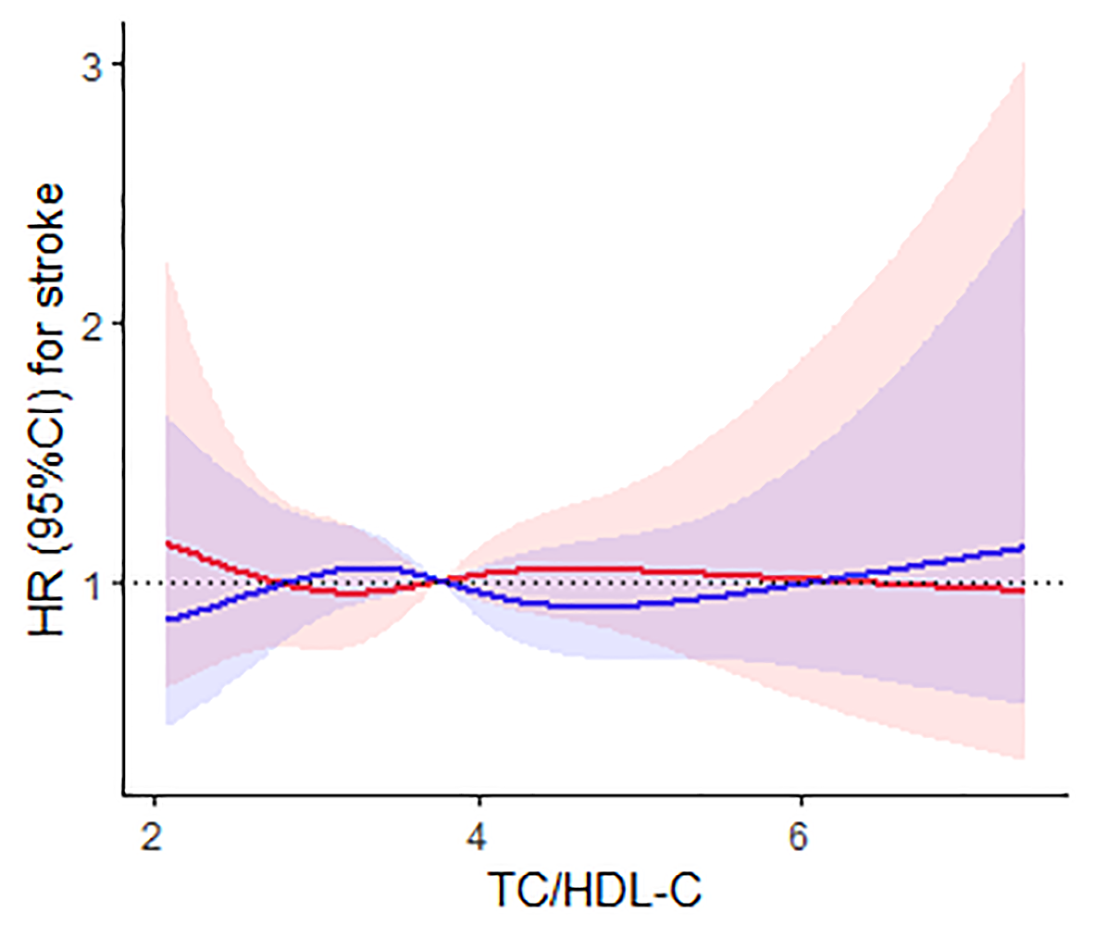

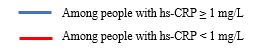


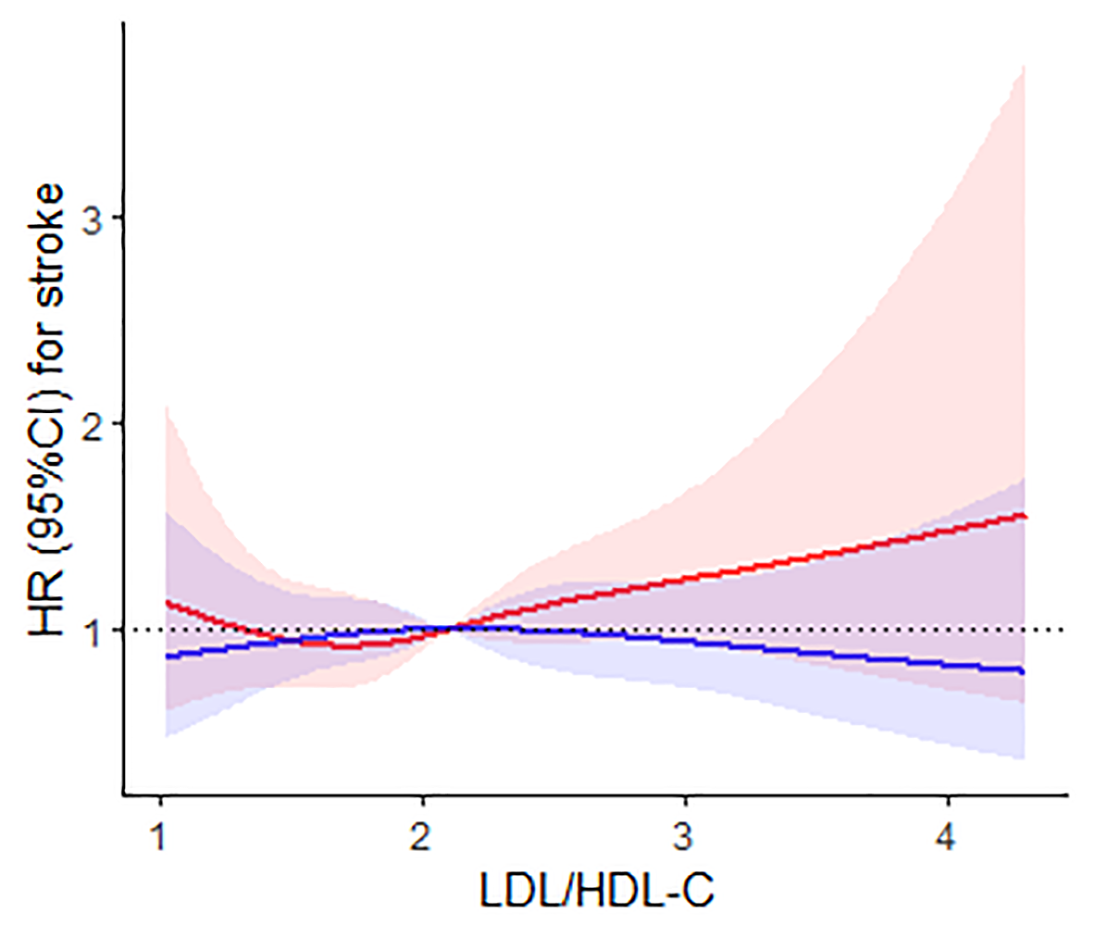

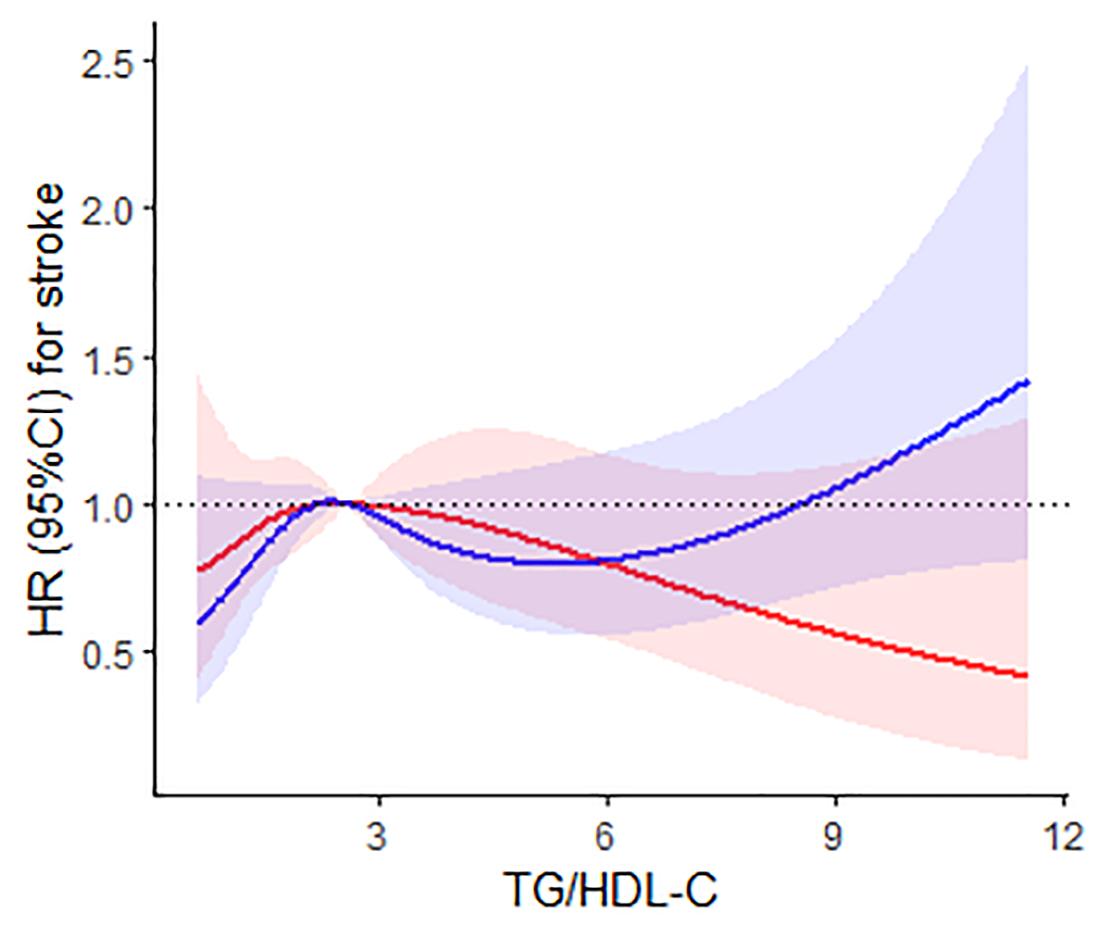


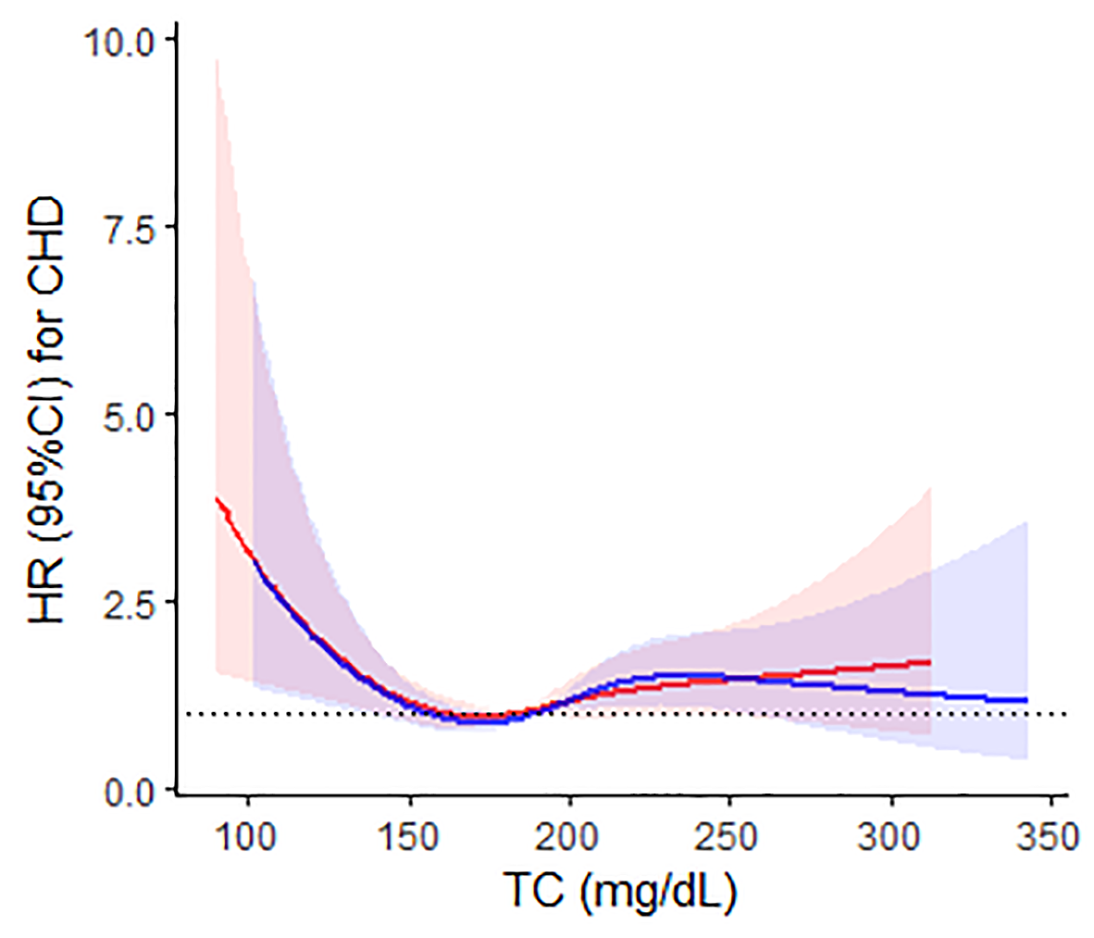

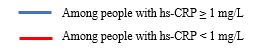


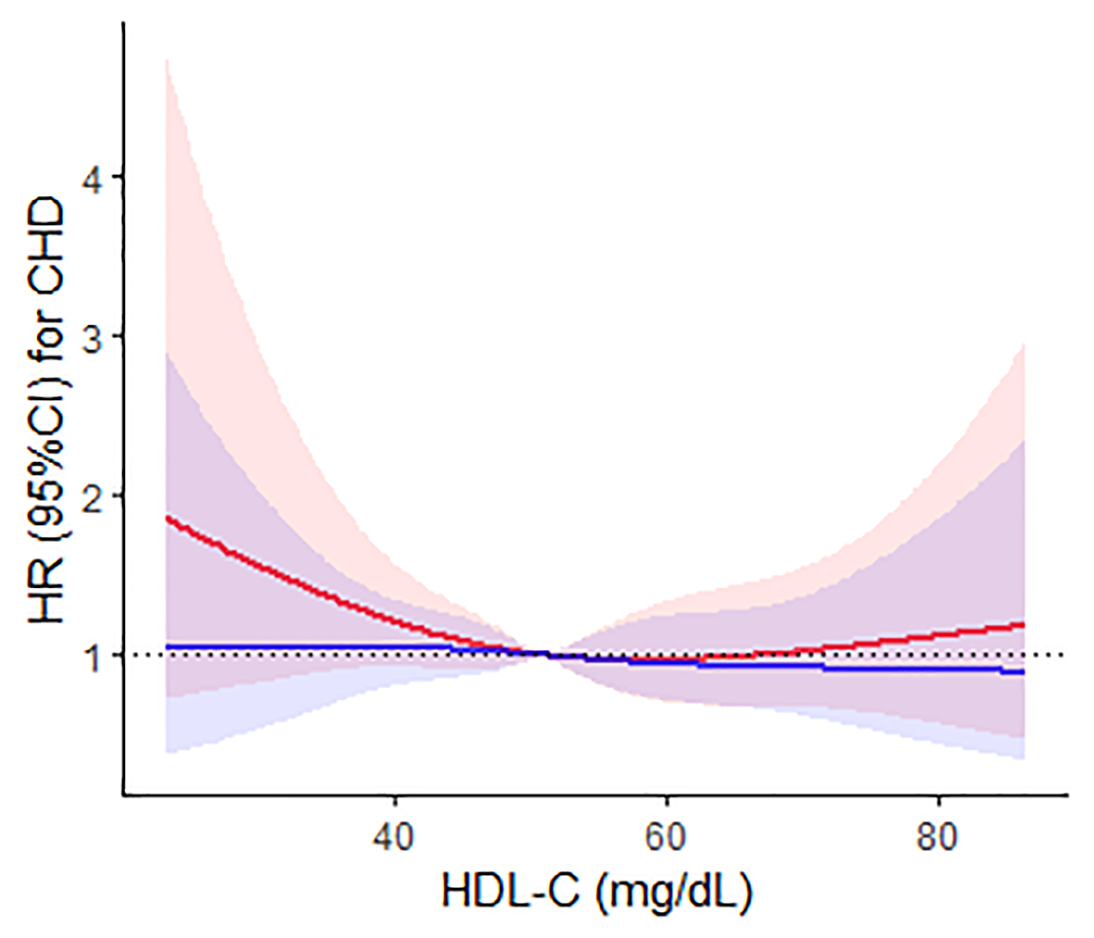

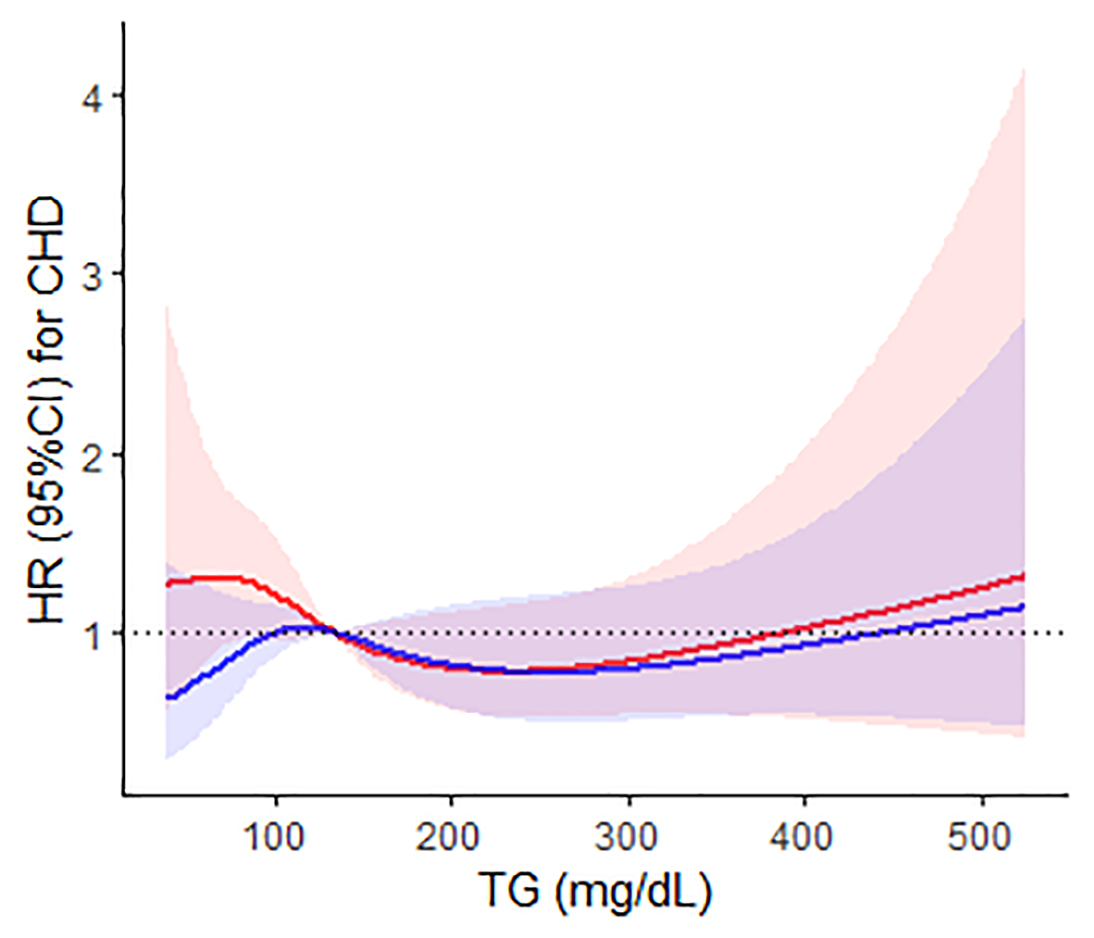


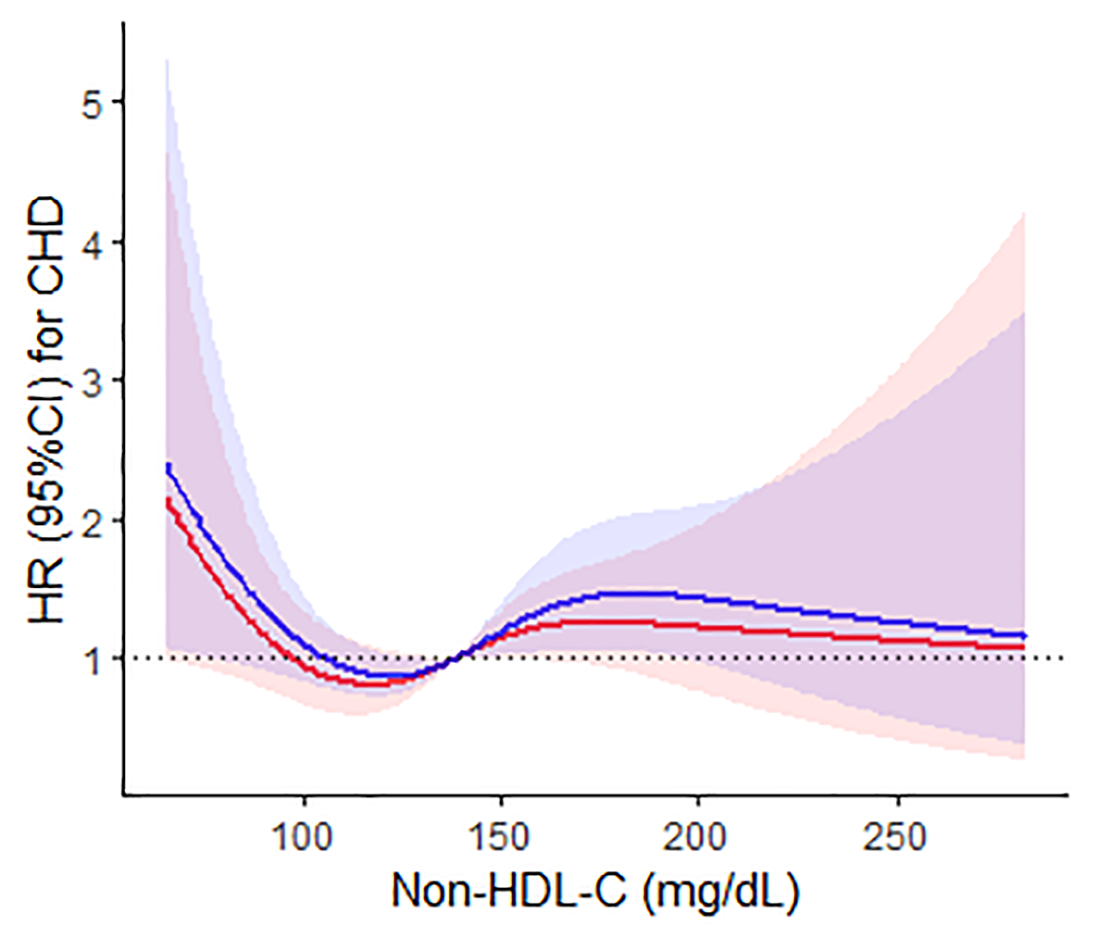

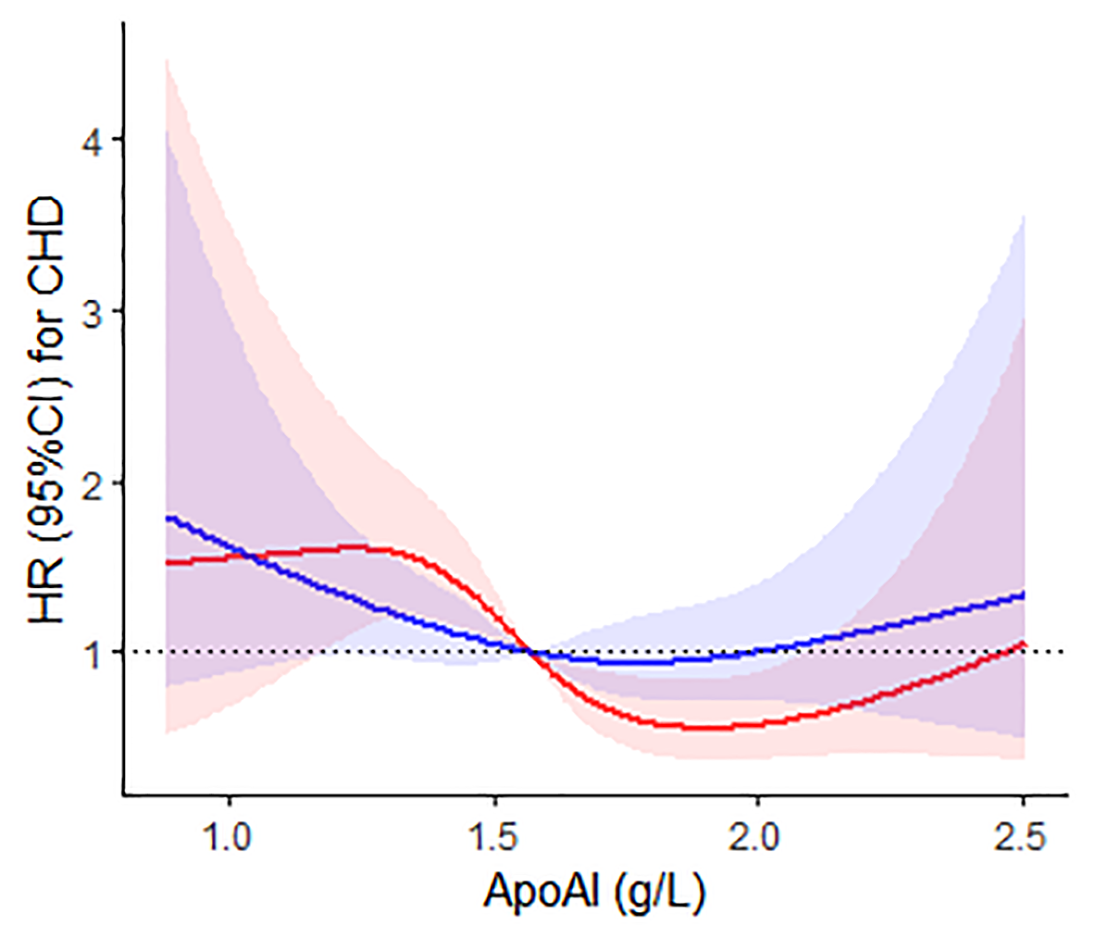

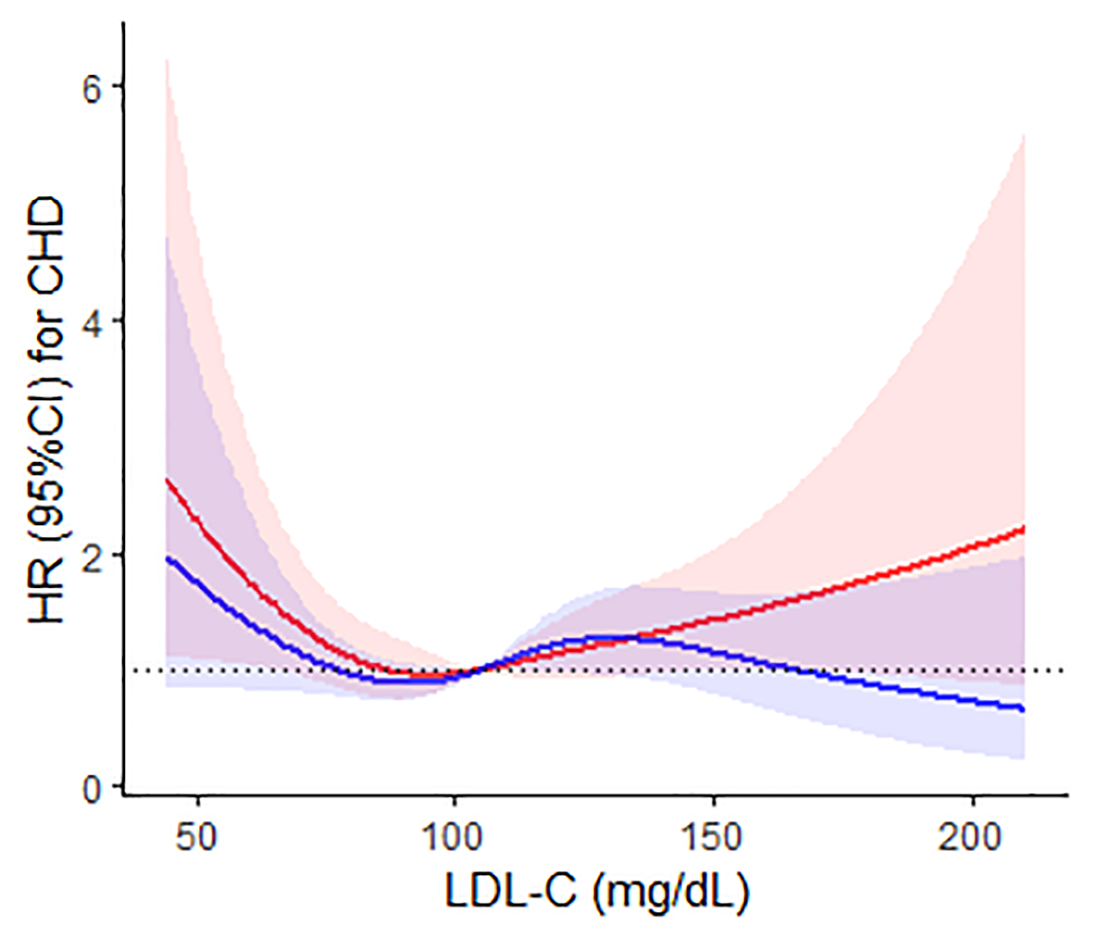


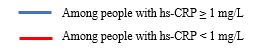


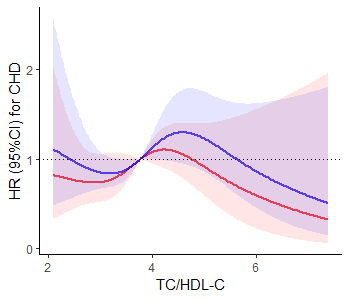

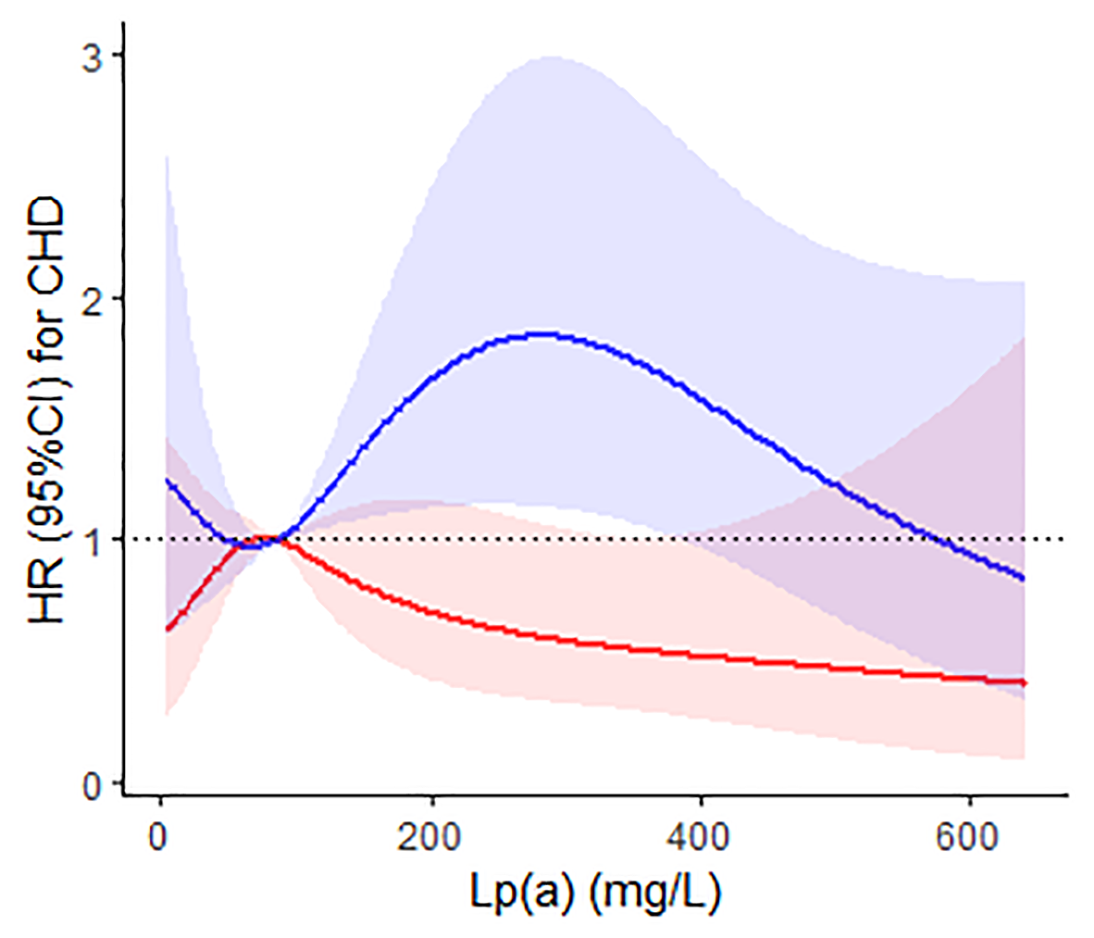

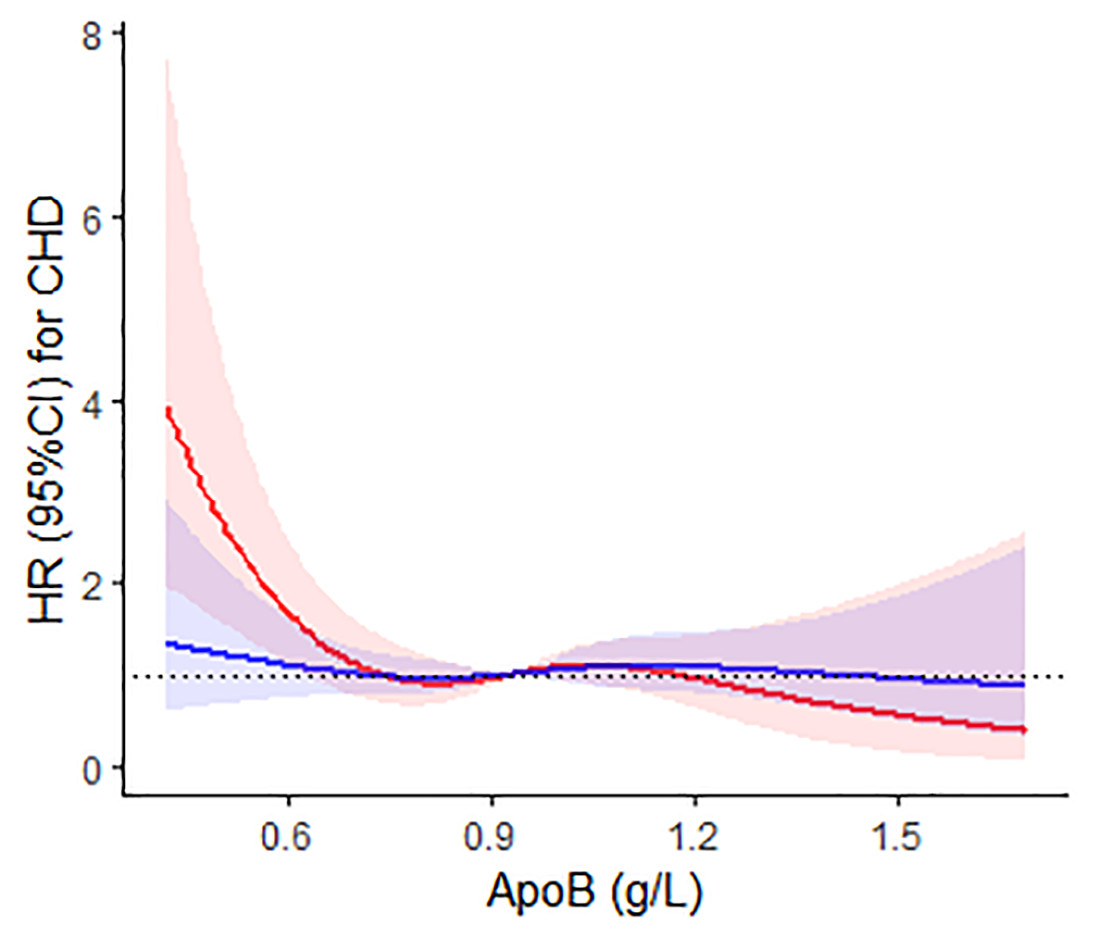


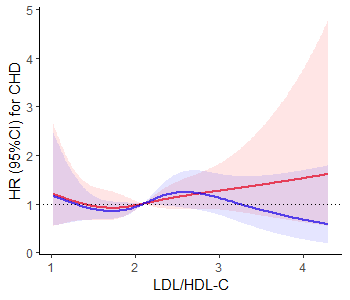

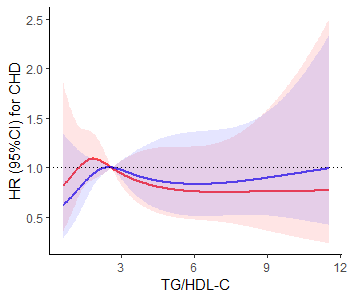


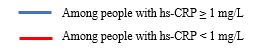


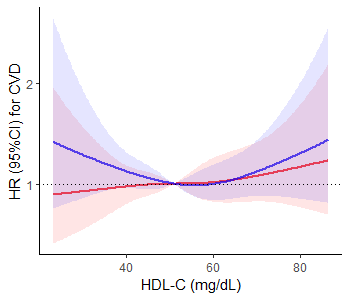

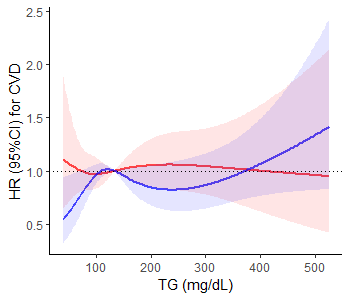

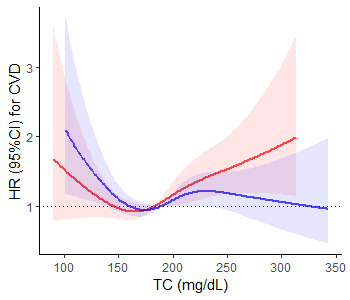


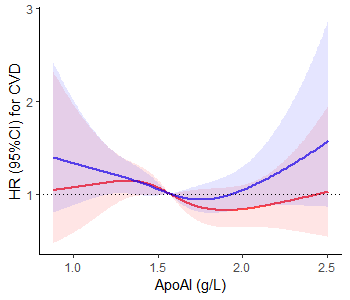

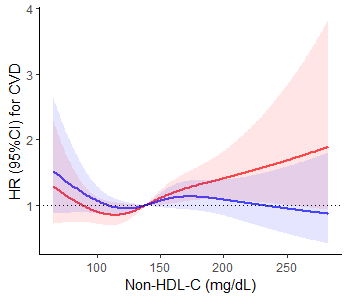

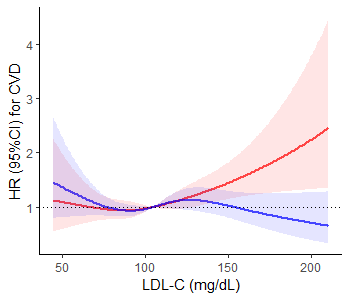


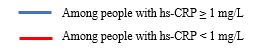


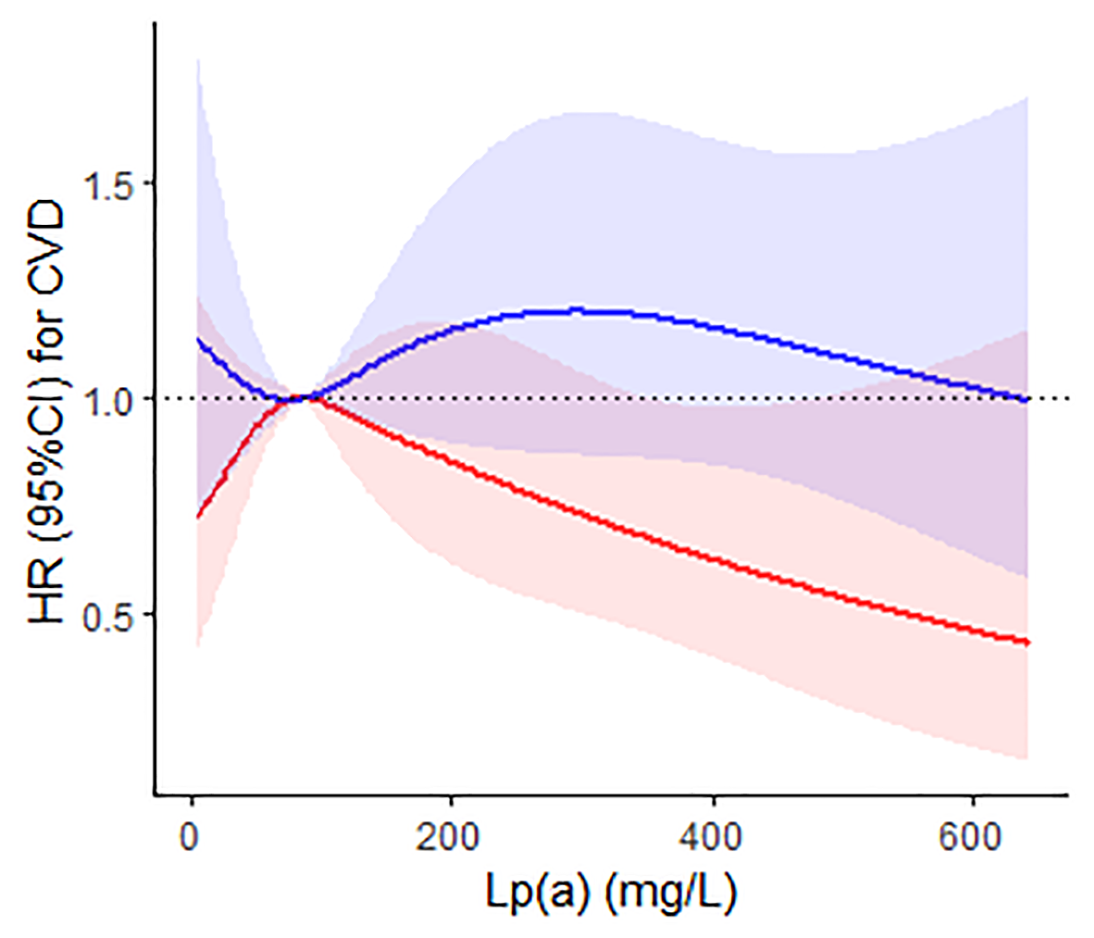

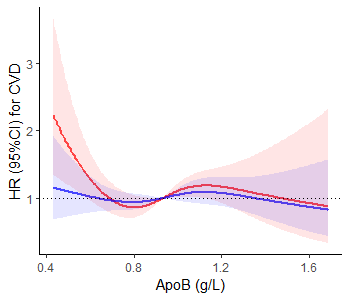

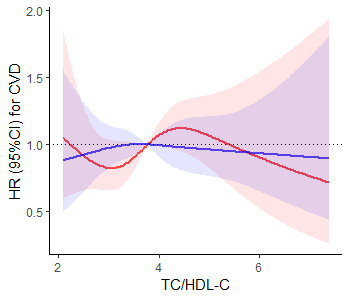


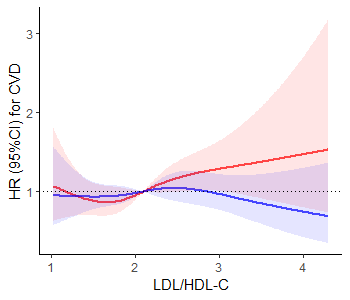

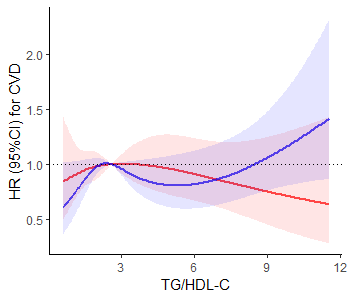


**Supplementary Fig. 1** Multiple restricted cubic splines regression analyses of lipids indices and risk of stroke, CHD, and CVD stratified by hs-CRP levels

Notes: Multiple restricted cubic splines (RCS) analyses with four knots at the 5th, 35th, 65th, and 95th percentiles. The models were adjusted for age, gender, smoking, drinking, hypertension, BMI, and diabetes. TG: triglycerides; TC: total cholesterol, HDL-C: high-density lipoprotein cholesterol, LDL-C: low-density lipoprotein cholesterol, BMI: body mass index, ApoAI: apolipoprotein AI, ApoB: apolipoprotein B, Lp(a): lipoprotein(a).

**Supplementary Table 8** P-values of multivariate restricted cubic splines regression analyses of lipids indices and risk of stroke, CHD, and CVD stratified by hs-CRP levels

|  |  | hs-CRP < 1 mg/L | |  | | hs-CRP ≥ 1 mg/L | | |  |
| --- | --- | --- | --- | --- | --- | --- | --- | --- | --- |
| Outcomes | Lipids | *P* for non-linear association | *P* for overall association | |  | | *P* for non-linear association | *P* for overall association | |
| Stroke | TC | 0.621 | 0.247 | |  | | 0.834 | 0.931 | |
|  | TG | 0.649 | 0.802 | |  | | 0.142 | 0.173 | |
|  | LDL-C | 0.742 | 0.070 | |  | | 0.716 | 0.839 | |
|  | HDL-C | 0.658 | 0.588 | |  | | 0.588 | 0.784 | |
|  | Non-HDL-C | 0.710 | 0.342 | |  | | 0.993 | 0.988 | |
|  | ApoAI | 0.975 | 0.968 | |  | | 0.560 | 0.715 | |
|  | ApoB | 0.013* | 0.033* | |  | | 0.603 | 0.793 | |
|  | Lp(a) | 0.521 | 0.589 | |  | | 0.915 | 0.946 | |
|  | TC/HDL-C | 0.658 | 0.838 | |  | | 0.865 | 0.962 | |
|  | TG/HDL-C | 0.390 | 0.405 | |  | | 0.158 | 0.152 | |
|  | LDL/HDL-C | 0.647 | 0.541 | |  | | 0.915 | 0.780 | |
| CHD | TC | 0.015* | 0.033* | |  | | 0.008* | 0.022* | |
|  | TG | 0.317 | 0.401 | |  | | 0.379 | 0.579 | |
|  | LDL-C | 0.034* | 0.076 | |  | | 0.126 | 0.236 | |
|  | HDL-C | 0.494 | 0.536 | |  | | 0.986 | 0.961 | |
|  | Non-HDL-C | 0.075 | 0.156 | |  | | 0.030* | 0.069 | |
|  | ApoAI | 0.059 | 0.004* | |  | | 0.366 | 0.332 | |
|  | ApoB | 0.021* | < 0.001* | |  | | 0.704 | 0.859 | |
|  | Lp(a) | 0.388 | 0.280 | |  | | 0.058 | 0.062 | |
|  | TC/HDL-C | 0.236 | 0.375 | |  | | 0.162 | 0.265 | |
|  | TG/HDL-C | 0.698 | 0.719 | |  | | 0.685 | 0.488 | |
|  | LDL/HDL-C | 0.667 | 0.648 | |  | | 0.243 | 0.407 | |
| CVD | TC | 0.077 | 0.020* | |  | | 0.028* | 0.060 | |
|  | TG | 0.867 | 0.963 | |  | | 0.070 | 0.062 | |
|  | LDL-C | 0.315 | 0.010* | |  | | 0.177 | 0.270 | |
|  | HDL-C | 0.954 | 0.861 | |  | | 0.416 | 0.246 | |
|  | Non-HDL-C | 0.178 | 0.050 | |  | | 0.237 | 0.393 | |
|  | ApoAI | 0.450 | 0.361 | |  | | 0.115 | 0.198 | |
|  | ApoB | 0.005* | 0.009* | |  | | 0.520 | 0.727 | |
|  | Lp(a) | 0.278 | 0.206 | |  | | 0.584 | 0.739 | |
|  | TC/HDL-C | 0.284 | 0.402 | |  | | 0.876 | 0.966 | |
|  | TG/HDL-C | 0.576 | 0.688 | |  | | 0.077 | 0.090 | |
|  | LDL/HDL-C | 0.190 | 0.476 | |  | | 0.703 | 0.506 | |

Notes: *: Significant at 0.05. TG: triglycerides; TC: total cholesterol, HDL-C: high-density lipoprotein cholesterol, LDL-C: low-density lipoprotein cholesterol, BMI: body mass index, ApoAI: apolipoprotein AI, ApoB: apolipoprotein B, Lp(a): lipoprotein(a).
